# Efficacy and Safety of Biologic Therapies for Uncontrolled Asthma: An Overview of Systematic Reviews

**DOI:** 10.1002/ppul.71727

**Published:** 2026-07-07

**Authors:** Ronaldo José Faria, Patrícia Silva Bazoni, Jéssica Barreto Ribeiro dos Santos, Erica Tatiane da Silva, Michael Ruberson Ribeiro da Silva

**Affiliations:** ^1^ Federal University of Espírito Santo, Postgraduate Program in Pharmaceutical Assistance Alegre Brazil; ^2^ Federal University of Espírito Santo, Group on Health Technology Assessment and Economy Alegre Brazil; ^3^ Program of Evidence for Health Policy and Technologies Oswaldo Cruz Foundation‐ Fiocruz Brasília Brasília Brazil

**Keywords:** evidence‐based medicine, monoclonal antibodies, precision medicine, severe asthma, type 2 inflammation

## Abstract

**Background:**

Asthma is a heterogeneous inflammatory disease that can cause substantial morbidity, reduced quality of life, and socioeconomic burden when uncontrolled. Biologic therapies have become central to the management of moderate‐to‐severe asthma by targeting specific inflammatory pathways.

**Methods:**

This overview of systematic reviews synthesized evidence on the efficacy and safety of biologic agents for uncontrolled asthma. Searches were conducted in Medline (PubMed), Embase, LILACS, Web of Science, and the Cochrane Library using MeSH and Emtree terms structured according to the PICOT framework. Systematic reviews with meta‐analyses or network meta‐analyses comparing omalizumab, mepolizumab, reslizumab, benralizumab, dupilumab, and tezepelumab were included.

**Results:**

In indirect comparisons, dupilumab and tezepelumab were associated with more favorable outcomes in reducing the annualized exacerbation rate (AER) than benralizumab, mepolizumab, and reslizumab in severe uncontrolled asthma. In eosinophilic asthma, indirect evidence suggested more favorable outcomes with mepolizumab, tezepelumab, and dupilumab across eosinophil‐defined subgroups (≥ 400 cells/μL, ≥ 300 cells/μL, ≥ 150 cells/μL, and < 150 cells/μL). In corticosteroid‐dependent asthma, benralizumab and dupilumab were associated with more favorable outcomes in selected comparisons. For lung function (FEV_1_), indirect evidence suggested more favorable outcomes with dupilumab. Regarding asthma control (ACQ), more favorable outcomes were observed with mepolizumab in specific comparisons. Regarding quality of life (AQLQ), more favorable outcomes were observed with omalizumab and tezepelumab in the general and eosinophilic asthma populations, respectively. One indirect comparison suggested lower odds of serious adverse events with mepolizumab.

**Conclusion:**

The available evidence suggests that biologic therapies may be effective and generally safe options for uncontrolled asthma, although the methodological quality of most included reviews was critically low. These findings support individualized treatment guided by biomarkers and clinical characteristics and highlight the need for standardized, methodologically rigorous future studies.

## 1 Introduction

Asthma is a heterogeneous chronic respiratory disease characterized by airway inflammation and variable symptoms, including wheezing, shortness of breath, chest tightness, and cough, which fluctuate in intensity over time. In some individuals, specific symptoms, such as cough, may predominate, and airflow limitation may become persistent. Although asthma is often associated with airway hyperresponsiveness and inflammation, these features are not required for diagnosis [1].

The Global Asthma Report (2022) estimates the global prevalence of asthma at 9.1% in children, 11.0% in adolescents, and 6.6% in adults [2]. Given its high prevalence, asthma imposes a substantial socioeconomic burden, especially when poorly controlled, leading to increased hospitalizations, school and work absenteeism, and a risk of death during exacerbations [1, 3, 4].

Asthma was historically regarded as a single, uniform disease; however, its clinical and biological heterogeneity is now well recognized. Variability in patient phenotypes, underlying immunopathological mechanisms (endotypes), and treatment responses underscores the need for a deeper understanding of disease subtypes to inform the development of more effective and personalized therapeutic strategies [5].

Patients with moderate‐to‐severe asthma account for much of the asthma‐related morbidity and mortality. In many cases, the disease remains uncontrolled despite optimal inhaled corticosteroid therapy. For these individuals, treatment with monoclonal antibodies has been shown to improve disease control and reduce asthma‐related morbidity and mortality [6].

Biologic agents have emerged as important therapeutic options for managing moderate‐to‐severe uncontrolled asthma. These include anti‐IgE (omalizumab), anti‐interleukin‐5 agents (mepolizumab, reslizumab, and benralizumab), anti‐interleukin‐4/interleukin‐13 therapy (dupilumab), and anti‐thymic stromal lymphopoietin therapy (tezepelumab) [7, 8].

In recent years, several studies have evaluated and compared the efficacy and safety of biologic therapies for asthma [9, 10, 11]. However, the volume and heterogeneity of the available evidence can hinder interpretation and limit its application in clinical practice.

In this context, overviews of systematic reviews have emerged as valuable tools for synthesizing findings across multiple studies and supporting evidence‐based decision‐making [12]. Accordingly, the present overview of systematic reviews aimed to synthesize the available evidence from systematic reviews comparing the efficacy and safety of biologic therapies for uncontrolled asthma.

## 2 Methods

### 2.1 Study Type

We conducted an overview of systematic reviews to synthesize evidence on the efficacy and safety of biologic therapies for uncontrolled asthma. We searched Medline (via PubMed), Embase, LILACS, Web of Science, and the Cochrane Library. The methodological steps included developing the search strategy, selecting studies, extracting data, and synthesizing the results. All stages were performed between July and November 2024.

This overview of systematic reviews was conducted and reported in accordance with the recommendations of the PRIOR statement for overviews of reviews.

### 2.2 Data Sources and Search Strategies

We developed the search strategy using appropriate indexing terms, including MeSH terms in PubMed and Emtree terms in Embase (details are available in the Material S1). The search was structured using the PICOT framework: “P” for population (patients aged ≥12 years diagnosed with uncontrolled asthma); “I” for intervention (biologic therapies); “C” for comparator (other biologic therapies); “O” for outcomes (efficacy, safety, lung function, and quality of life); and “T” for type of study (systematic reviews with meta‐analyses). Based on this framework, we formulated the following research question: *Is there a difference in efficacy and safety among biologic therapies for the treatment of uncontrolled asthma?*

### 2.3 Study Search Process and Selection

We conducted the study selection process using the Rayyan QCRI platform [13]. Two independent reviewers screened each article, and any disagreements were resolved by a third reviewer. Data extraction was performed in duplicate by two independent researchers. The following data were extracted from the included reviews: author, year of publication, number of included studies, population characteristics, biologic therapies evaluated, outcomes assessed, and main efficacy and safety findings. A qualitative synthesis of the findings was performed, focusing on comparative efficacy and safety‐related outcomes across biologic therapies. No additional sensitivity analyses were performed.

### 2.4 Eligibility Criteria

This overview included only systematic reviews with meta‐analyses or network meta‐analyses that performed direct or indirect comparisons between biologic therapies used to treat uncontrolled asthma. There were no restrictions regarding publication date or language. The biologic agents evaluated were omalizumab, mepolizumab, reslizumab, benralizumab, dupilumab, and tezepelumab.

For the purposes of this overview, systematic reviews were defined as studies reporting a structured literature search, explicit eligibility criteria, and a systematic synthesis of evidence, with or without quantitative meta‐analysis.

Potential overlap among primary studies across the included systematic reviews was qualitatively assessed and considered during data interpretation, given that overviews of reviews may include the same clinical trials in multiple evidence syntheses. This overlap may influence the weight of the evidence and lead to duplication of findings across reviews. However, a formal quantitative assessment of overlap, such as the corrected covered area (CCA), was not performed. To minimize inconsistencies, the included reviews were carefully screened, and data extraction focused on identifying convergence and divergence of results across studies.

### 2.5 Evaluation of the Methodological Quality of the Studies

We assessed the methodological quality of the included systematic reviews using the AMSTAR 2 (A Measurement Tool to Assess Systematic Reviews) checklist.

### 2.6 Review Registration

This overview of systematic reviews was prospectively registered in the Open Science Framework (OSF) under DOI: 10.17605/OSF.IO/FKDRA.

## 3 Results

**Figure 1:**
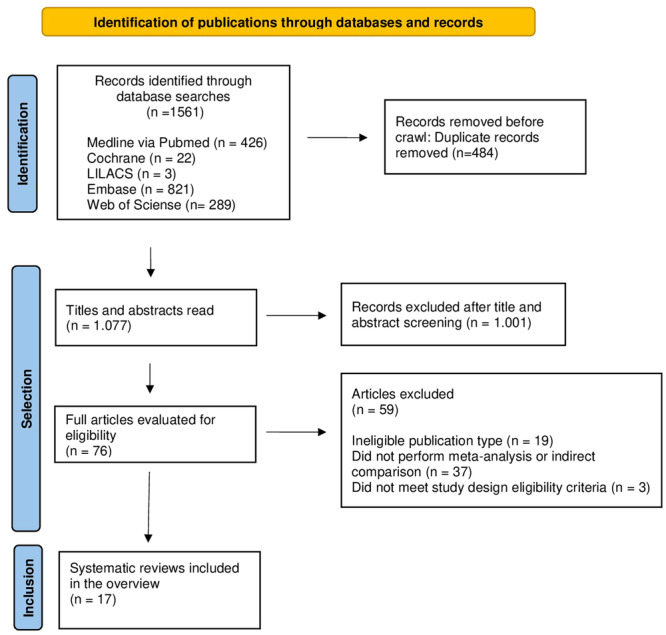
PRISMA flow diagram of study identification, screening, eligibility, and inclusion. [Color figure can be viewed at wileyonlinelibrary.com]

The electronic database search initially identified 1561 records. After 484 duplicates were removed, 1077 studies were screened by title and abstract. Of these, 1001 were excluded because they did not meet the study objectives or scope. A total of 76 articles were selected for full‐text review, and 59 were subsequently excluded for failing to meet the eligibility criteria. Ultimately, 17 systematic reviews were included in this overview (Figure 1), and their main characteristics are summarized in Table 1. The criteria used to exclude studies during full‐text screening are detailed in the Material S1.

**Table 1:** Key characteristics of the systematic reviews included in the overview.

Study	Objective	Population	Methodology	Biologic agents evaluated	No. of Studies	Outcomes
Phinyo et al. [11]	To evaluate the comparative efficacy and safety in patients dependent on oral corticosteroids (OCS).	Patients with OCS‐dependent asthma	Systematic review and network meta‐analysis	Tezepelumab, Dupilumab, Benralizumab, Reslizumab and Mepolizumab	6 (Tezepelumab: 1, Dupilumab: 1, Benralizumab: 1, Reslizumab: 1, Mepolizumab: 2)	Primary: OCS dose reduction; Secondary: Exacerbation rate
Akenroye et al. [10]	To compare the safety and efficacy in patients with severe eosinophilic asthma.	Patients ≥ 12 years of age with severe asthma	Systematic review and network meta‐analysis	Mepolizumab, Dupilumab and Benralizumab	8 (Mepolizumab: 3, Dupilumab: 2, Benralizumab: 3)	Primary: Exacerbation rate; Secondary: Pulmonary function (FEV1), Symptom control (ACQ), Adverse events
Nopsopon et al. [6]	To compare tezepelumab with other biologics for treating eosinophilic asthma.	Patients ≥ 12 years of age with severe eosinophilic asthma	Systematic review and network meta‐analysis	Tezepelumab, Mepolizumab, Benralizumab and Dupilumab	10 (Tezepelumab: 2, Mepolizumab: 3, Benralizumab: 3, Dupilumab: 2)	Primary: Exacerbation rate, Pulmonary function (FEV1), Symptom control (ACQ)
Ando et al. [9]	To compare the efficacy of biologics based on type 2 inflammatory biomarkers.	Patients ≥ 12 years of age with uncontrolled asthma	Systematic review and network meta‐analysis	Tezepelumab, Dupilumab, Mepolizumab and Benralizumab	8 (Tezepelumab: 1, Dupilumab: 1, Mepolizumab: 2, Benralizumab: 4)	Primary: Exacerbation rate, Adverse events; Secondary: Pulmonary function (FEV1), Symptom control (ACQ), Quality of life (AQLQ)
Bateman et al. [14]	To compare dupilumab with anti‐IL‐5 and anti‐IgE biologics.	Patients ≥ 12 years of age with persistent/uncontrolled asthma	Systematic review and network meta‐analysis	Dupilumab, Reslizumab, Mepolizumab, Benralizumab and Omalizumab	13 (Dupilumab: 2, Reslizumab: 5, Mepolizumab: 3, Benralizumab: 2, Omalizumab: 1)	Exacerbation rate, Pulmonary function (FEV1)
Menzies‐Gow et al. [15]	To compare tezepelumab with other biologic medicines.	Patients ≥ 12 years of age with severe, uncontrolled asthma	Systematic review with network meta‐analysis	Tezepelumab, Omalizumab, Dupilumab, Reslizumab, Mepolizumab and Benralizumab	17 (Tezepelumab: 2, Dupilumab: 3, Omalizumab: 5, Reslizumab: 2, Mepolizumab: 2, Benralizumab: 3)	Exacerbation rate (overall and requiring hospitalization or emergency room visits);
Bourdin et al. [16]	To indirectly compare dupilumab, mepolizumab, and benralizumab.	Patients with asthma	Systematic review with network meta‐analysis	Dupilumab, Mepolizumab and Benralizumab	3 (Dupilumab: 1, Mepolizumab: 1, Benralizumab: 1)	Exacerbation rate, Symptom control (ACQ), Oral corticosteroid (OCS) dose
Ando et al. [17]	To indirectly compare dupilumab and benralizumab.	Patients with asthma	Systematic review with network meta‐analysis	Dupilumab and Benralizumab	2 (Dupilumab: 1, Benralizumab: 1)	Primary: Exacerbation rate; Secondary: Pulmonary function (FEV1), Quality of life (AQLQ), Adverse events
Ramonell & Iftikhar. [18]	To indirectly compare dupilumab, mepolizumab, benralizumab, and reslizumab.	Patients ≥ 12 years of age with eosinophilic asthma	Systematic review with network meta‐analysis	Dupilumab, Mepolizumab, Benralizumab and Reslizumab	9 (Dupilumab: 4, Mepolizumab: 2, Benralizumab: 2, Reslizumab: 1)	Exacerbation rate
Edris et al. [19]	To indirectly compare reslizumab, mepolizumab, benralizumab, dupilumab, and tezepelumab.	Patients with asthma	Systematic review with network meta‐analysis	Reslizumab, Mepolizumab, Benralizumab, Dupilumab and Tezepelumab	19 (Dupilumab: 3, Mepolizumab: 5, Benralizumab: 6, Reslizumab: 4 Tezepelumab:1)	Exacerbation rate
Busse et al. [20]	To indirectly compare reslizumab, mepolizumab, and benralizumab.	Patients ≥ 12 years of age with severe eosinophilic asthma	Systematic review with network meta‐analysis	Reslizumab, Mepolizumab and Benralizumab	9 (Reslizumab: 5, Mepolizumab: 2, Benralizumab: 2)	Exacerbation rate (overall and requiring hospitalization or emergency room visits); Pulmonary function (FEV1); Symptom control score (ACQ)
Yan et al. [21]	To indirectly compare reslizumab and mepolizumab.	Patients ≥ 12 years of age with severe, uncontrolled asthma	Systematic review with network meta‐analysis	Reslizumab and Mepolizumab	8 (Reslizumab: 5, Mepolizumab: 3)	Primary: Severe exacerbation and exacerbation rate; Secondary: Time to first exacerbation, change in FEV_1_ and FEV_1_% predicted, change in eosinophil count, adverse events (total and severe), discontinuations due to adverse events
Iftikhar et al. [22]	To indirectly compare dupilumab, mepolizumab, benralizumab, and reslizumab.	Patients with asthma	Systematic review with network meta‐analysis	Dupilumab, Mepolizumab, Benralizumab and Reslizumab	19 (Dupilumab: 2, Benralizumab: 7, Mepolizumab: 6, Reslizumab: 4)	Pulmonary function (FEV1); Quality of life (AQLQ); Symptom control score (ACQ)
Nachef et al. [23]	To indirectly compare mepolizumab and omalizumab.	Patients ≥ 12 years of age with symptomatic asthma	Systematic review with meta‐analysis	Mepolizumab and Omalizumab	22 (Mepolizumab: 4, Omalizumab: 18)	Pulmonary function test (FEV1), Quality of life (AQLQ), Symptom control (ACQ), or Adverse event rates
Bourdin et al. [24]	To indirectly compare reslizumab with mepolizumab and benralizumab.	Patients ≥ 18 years of age with severe asthma	Systematic review with meta‐analysis	Reslizumab, Mepolizumab and Benralizumab	8 (Reslizumab: 2, Mepolizumab: 3, Benralizumab: 3)	Exacerbation rate (overall and requiring hospitalization or emergency room visits); Pulmonary function (FEV1)
Henriksen et al. [25]	To indirectly compare reslizumab and mepolizumab.	Patients ≥ 18 years of age with severe eosinophilic asthma	Systematic review with network meta‐analysis	Mepolizumab and Reslizumab	9 (Mepolizumab: 4, Reslizumab: 5)	Pulmonary function (FEV1); Quality of life (AQLQ); Symptom control (ACQ)
Cockle et al. [26]	To indirectly compare mepolizumab and omalizumab.	Patients ≥ 12 years of age with severe asthma	Systematic review with network meta‐analysis	Mepolizumab and Omalizumab	4 (Mepolizumab: 1, Omalizumab: 3)	Primary: Exacerbation rate (overall and requiring hospitalization or emergency room visits); Secondary: Pulmonary function (FEV1); Adverse events; Serious adverse events

*Source:* Authors.

The analyses covered multiple clinical outcomes and provided indirect comparisons among biologic therapies for moderate‐to‐severe uncontrolled asthma. The main clinically relevant findings are presented below in an integrated manner, whereas complete results, including non‐significant comparisons, are provided in the Material S1.

### 3.1 Annual Exacerbation Rate (AER)

#### 3.1.1 General Population With Severe Uncontrolled Asthma

This outcome was evaluated in six studies (Ando et al. [9]; Bateman et al. [14]; Menzies‐Gow et al. [15]; Ando et al. [17]; Ramonell & Iftikhar [18]; Bourdin et al. [24]), encompassing multiple indirect comparisons summarized in Table 2. In the study by Ando et al. [9], indirect comparisons suggested that tezepelumab may be associated with greater reductions in exacerbations than benralizumab (RR = 0.73; 95% CI: 0.59–0.92). Additionally, indirect evidence suggested less favorable results with benralizumab relative to mepolizumab (RR = 1.35; 95% CI: 1.05–1.73). Bateman et al. [14] reported indirect comparisons suggesting more favorable outcomes with dupilumab than with benralizumab (RR = 0.46; 95% CI: 0.32–0.67), mepolizumab (RR = 0.72; 95% CI: 0.57–0.92), and reslizumab (RR = 0.62; 95% CI: 0.48–0.79) (Table 2).

**Table 2:** Statistically significant indirect comparisons of biologic therapies for annual exacerbation rate (AER) in the general population and oral corticosteroid (OCS)‐dependent asthma.

Comparison	No. of studies performing the comparison	No. of studies with significant results	Result (RR, 95% CI)	Favored drug
**General population**				
Dupilumab versus Benralizumab	5	1	Bateman et al. [14]: RR = 0.46 (0.32–0.67)	Dupilumab
Dupilumab versus Mepolizumab	4	1	Bateman et al. [14]: RR = 0.72 (0.57–0.92)	Dupilumab
Dupilumab versus Reslizumab	3	1	Bateman et al. [14]: RR = 0.62 (0.48–0.79)	Dupilumab
Benralizumab versus Mepolizumab	3	1	Ando et al. [9]: RR = 1.35 (1.05–1.73)	Mepolizumab
Tezepelumab versus Benralizumab	1	1	Ando et al. [9]: RR = 0.73 (0.59–0.92)	Tezepelumab
**Oral Corticosteroid (OCS)‐Dependent Asthma**				
Benralizumab versus Mepolizumab	2	1	Phinyo et al. [11]: RR = 0.44 (0.22–0.87)	Benralizumab
Benralizumab versus Tezepelumab	1	1	Phinyo et al. [11]: RR = 0.43 (0.21–0.90)	Benralizumab
Benralizumab versus Reslizumab	1	1	Phinyo et al. [11]: RR = 0.37 (0.17–0.77)	Benralizumab
Dupilumab versus Reslizumab	1	1	Phinyo et al. [11]: RR = 0.50 (0.26–0.95)	Dupilumab

*Source:* Authors. RR = Relative Risk. The annual exacerbation rate (AER) was evaluated across six studies: Ando et al. [9], Bateman et al. [14], Menzies‐Gow et al. [15], Ando et al. [17], Ramonell & Iftikhar [18], and Bourdin et al. [24]. The annual exacerbation rate among patients with oral corticosteroid (OCS)‐dependent asthma was evaluated in two studies: Phinyo et al. [11] and Bourdin et al. [16]. Comparisons without statistically significant differences are further detailed in the Material S1.

#### 3.1.2 Oral Corticosteroid (OCS)‐Dependent Asthma

Two studies (Phinyo et al. [11]; Bourdin et al. [16]) evaluated this outcome in this patient subgroup, encompassing the comparisons summarized in Table 2. Phinyo et al. [11] reported indirect comparisons suggesting more favorable outcomes with benralizumab administered every 8 weeks (Q8W) than with mepolizumab (RR = 0.44; 95% CI: 0.22–0.87), tezepelumab (RR = 0.43; 95% CI: 0.21–0.90), and reslizumab (RR = 0.37; 95% CI: 0.17–0.77). Indirect evidence also suggested more favorable results with dupilumab than with reslizumab (RR = 0.50; 95% CI: 0.26–0.95) (Table 2).

#### 3.1.3 Eosinophilic Asthma

This outcome was analyzed across eosinophilic asthma subgroups, suggesting different efficacy patterns among the evaluated biologics. Multiple indirect comparisons are detailed in Table 3.

**Table 3:** Statistically significant indirect comparisons of biologic therapies for annual exacerbation rate (AER) in eosinophilic asthma.

Eosinophil subgroup	Comparison	No. of studies performing the comparison	No. of studies with significant results	Result (RR 95% CI)	Favored drug
≥ 400 cells/μL	Mepolizumab versus Benralizumab	1	1	Busse et al. [20]: RR = 0.55 (0.35–0.87)	Mepolizumab
	Mepolizumab versus Reslizumab	1	1	Busse et al. [20]: RR = 0,55 (0.36–0.85)	Mepolizumab
≥ 300 cells/μL	Dupilumab versus Benralizumab	5	3	Ando et al. [17]: RR = 0.58 (0.39–0.84)	Dupilumab
				Akenroye et al. [10]: RR = 0.66 (0.47–0.94)	Dupilumab
				Ando et al. [9]: RR = 0.56 (0.38–0.82)	Dupilumab
	Mepolizumab versus Benralizumab	4	2	Akenroye et al. [10]: RR = 0.75 (0.60–0.95)	Mepolizumab
				Busse et al. [20]: RR = 0.61 (0.37–0.99)	Mepolizumab
	Reslizumab versus Mepolizumab	3	1	Yan et al. [21]: RR = 0.70 (0.53–0.95)	Reslizumab
	Tezepelumab versus Benralizumab	3	2	Nopsopon et al. [6]: RR = 0.63 (0.46–0.86)	Tezepelumab
				Ando et al. [9]: RR = 0.51 (0.36–0.72)	Tezepelumab
	Benralizumab versus Mepolizumab	3	1	Ando et al. [9]: RR = 1.89 (1.18–3.03)*	Mepolizumab
≥150 cells/μL	Dupilumab versus Benralizumab	4	2	Ando et al. [17]: RR = 0.51 (0.29–0.92)	Dupilumab
				Ando et al. [9]: RR = 0,67 (0,49–0.91)	Dupilumab
	Mepolizumab versus Benralizumab	3	2	Menzies‐Gow et al. [15]: RR = 0,68 (0,50–0.92)	Mepolizumab
				Busse et al. [20]: RR = 0.66 (0.49–0.89)	Mepolizumab
	Tezepelumab versus Benralizumab	2	2	Menzies‐Gow et al. [15]: RR = 0.63 (0.49–0.82)	Tezepelumab
				Ando et al. [9]: RR = 0,66 (0.51–0.85)	Tezepelumab
	Benralizumab versus Mepolizumab	2	1	Ando et al. [9]: RR = 1.52 (1.03–2.24)*	Mepolizumab
	Tezepelumab versus Omalizumab	1	1	Menzies‐Gow et al. [15]: RR = 0.63 (0.43–0.94)	Tezepelumab
<150 cells/μL	Tezepelumab versus Dupilumab	2	2	Ando et al. [9]: RR = 0.53 (0.30–0.94)	Tezepelumab
				Menzies‐Gow et al.[15]: RR = 0.48 (0.28–0.84)	Tezepelumab
					

*Source:* Authors. RR = Relative Risk. The annual exacerbation rate (AER) for eosinophilic asthma was analyzed across subgroups defined by eosinophil thresholds (≥400, ≥300, ≥150, and <150 cells/μL). Studies evaluating each subgroup: ≥400 cells/μL: Busse et al. [20]. ≥300 cells/μL: Busse et al. [20]; Edris et al. [19]; Ando et al. [17]; Menzies‐Gow et al. [15]; Akenroye et al. [10]; Nopsopon et al. [6]; Ando et al. [9]; Bateman et al. [14]; Cockle et al. [26]; Yan et al. [21]. ≥150 cells/μL: Busse et al. [20]; Ando et al. [17]; Menzies‐Gow et al. [15]; Akenroye et al. [10]; Ando et al. [9]. <150 cells/μL: Ando et al. [17]; Menzies‐Gow et al. [15]; Ando et al. [9]. Comparisons without statistically significant differences are further detailed in the Material S1.

* Indicates lower efficacy of Benralizumab compared with Mepolizumab.

**Eosinophils** ≥ **400 cells/μL**: Busse et al. [20] evaluated mepolizumab versus benralizumab, mepolizumab versus reslizumab, and reslizumab versus benralizumab. Indirect comparisons suggested more favorable results with mepolizumab than with benralizumab and reslizumab (RR = 0.55; 95% CI: 0.35–0.87 and RR = 0.55; 95% CI: 0.36–0.85, respectively).

**Eosinophils** ≥ **300 cells/μL**: This outcome was assessed in ten studies (Busse et al. [20], 2019; Edris et al. [19]; Ando et al. [17]; Menzies‐Gow et al. [15]; Akenroye et al. [10]; Nopsopon et al. [6]; Ando et al. [9]; Bateman et al. [14]; Cockle et al. [26]; Yan et al. [21]). Indirect comparisons suggested more favorable results with dupilumab than with benralizumab in Ando et al. [17] (RR = 0.58; 95% CI: 0.39–0.84), Akenroye et al. [10] (RR = 0.66; 95% CI: 0.47–0.94), and Ando et al. [9] (RR = 0.56; 95% CI: 0.38–0.82). Similar findings were observed for tezepelumab compared with benralizumab in Nopsopon et al. [6] (RR = 0.63; 95% CI: 0.46–0.86) and Ando et al. [9] (RR = 0.51; 95% CI: 0.36–0.72). Evidence supporting more favorable comparative outcomes with mepolizumab than benralizumab was reported by Busse et al. [20] (RR = 0.61; 95% CI: 0.37–0.99), Akenroye et al. [10] (RR = 0.75; 95% CI: 0.60–0.95), and Ando et al. [9] (RR = 1.89; 95% CI: 1.18–3.03). Finally, Yan et al. [21] reported more favorable results with reslizumab than with mepolizumab (RR = 0.70; 95% CI: 0.53–0.95).

**Eosinophils** ≥ **150 cells/μL:** Five studies contributed to this subgroup analysis (Busse et al. [20]; Ando et al. [17]; Menzies‐Gow et al. [15]; Akenroye et al. [10]; Ando et al. [9]). In two investigations by Ando et al. [9, 17], indirect comparisons suggested more favorable outcomes with dupilumab than with benralizumab, with statistically significant reductions in exacerbation risk (RR = 0.51; 95% CI: 0.29–0.92; and RR = 0.67; 95% CI: 0.49–0.91, respectively). Indirect evidence suggested more favorable results with mepolizumab than with benralizumab in Busse et al. [20] (RR = 0.66; 95% CI: 0.49–0.89) and Menzies‐Gow et al. [15] (RR = 0.68; 95% CI: 0.50–0.92). Ando et al. [9] further reported less favorable results with benralizumab relative to mepolizumab (RR = 1.52; 95% CI: 1.03–2.24) and more favorable results with tezepelumab relative to benralizumab (RR = 0.66; 95% CI: 0.51–0.85). Menzies‐Gow et al. [15] reported indirect comparisons suggesting more favorable outcomes with tezepelumab than with omalizumab (RR = 0.63; 95% CI: 0.43–0.94) and benralizumab (RR = 0.63; 95% CI: 0.49–0.82), suggesting a consistent pattern of findings in this subgroup.

**Eosinophils** < **150 cells/μL:** This subgroup was assessed in three studies (Ando et al. [17]; Menzies‐Gow et al. [15]; Ando et al. [9]). Indirect comparisons suggested more favorable results with tezepelumab than with dupilumab in Ando et al. [9] (RR = 0.53; 95% CI: 0.30–0.94) and Menzies‐Gow et al. [15] (RR = 0.48; 95% CI: 0.28–0.84).

### 3.2 Pulmonary Function—Change in FEV_1_ (Liters)

#### 3.2.1 General Population With Severe Uncontrolled Asthma

Six studies (Ando et al. [9]; Bateman et al. [14]; Nachef et al. [23]; Ando et al. [17]; Bourdin et al. [24]; Henriksen et al. [25]) evaluated this outcome in the general population with severe uncontrolled asthma, including multiple comparisons summarized in Table 4. Bateman et al. [14] reported a statistically significant improvement in FEV_1_ with dupilumab compared with benralizumab, both at Week 12 (MD = 0.12 L; 95% CI: 0.02–0.22) and at Week 24 (MD = 0.11 L; 95% CI: 0.01–0.21). At Week 24, indirect comparisons also suggested more favorable results with dupilumab than with reslizumab (MD = 0.14 L; 95% CI: 0.04–0.24), whereas the other comparisons did not show statistically significant differences (Table 4).

**Table 4:** Pulmonary function (FEV_1_, L): statistically significant indirect comparisons in the general population and eosinophilic asthma.

General population					
Comparison	No. of studies performing the comparison	No. of studies with significant results	Result (MD, 95% CI)	Favored drug	
Dupilumab versus Benralizumab (Week 12)	1	1	Bateman et al. [14]: MD = 0.12 (0.02–0.22)	Dupilumab	
					
Dupilumab versus Benralizumab (Week 24)	1	1	Bateman et al. [14]: MD = 0.11 (0.01–0.21)	Dupilumab	
					
Dupilumab versus Reslizumab (Week 24)	1	1	Bateman et al. [14]: MD = 0.14 (0.04–0.24)	Dupilumab	
					

Eosinophilic Asthma					
**Eosinophil threshold**	**Comparison**	**No. of studies performing the comparison**	**No. of studies with significant results**	**Result (MD, 95% CI)**	**Favored drug**
≥400 cells/μL	Benralizumab versus Reslizumab	1	1	Busse et al. [20]: MD = 0.11 (0.01–0.20)	Benralizumab
					
≥300 cells/μL	Dupilumab versus Benralizumab	4	1	Ando et al. [9]: MD = 0.12 (0.02–0.20)	Dupilumab
					
	Tezepelumab versus Benralizumab	2	1	Ando et al. [9]: MD = 0.10 (0.01–0.19)	Tezepelumab
					
	Reslizumab versus Mepolizumab	2	1	Yan et al. [21]: W4 MD = 0.14 (0.03–0.24)	Reslizumab
					

*Source:* Authors; MD = Mean Difference; CI = Confidence Interval; W = Week The change in FEV_1_ was analyzed across six studies in patients with severe uncontrolled asthma (Ando et al. [9]; Bateman et al. [14]; Nachef et al. [23]; Ando et al. [17]; Bourdin et al. [24]; Henriksen et al. [25]). The change in FEV_1_ (L) for eosinophilic asthma was evaluated in subgroups defined by eosinophil count thresholds (≥ 400 cells/μL and ≥ 300 cells/μL). Studies evaluating each subgroup: ≥ 400 cells/μL: Busse et al. [20]. ≥ 300 cells/μL: Busse et al. [20]; Cockle et al. [26]; Ando et al. [17]; Yan et al. [21]; Akenroye et al. [10]; Nopsopon et al. [6]; Ando et al. [9]; Bateman et al. [14]; Iftikhar et al. [22]. Significant results reported as percent predicted FEV_1_ are described in the text. Comparisons without statistically significant differences are further detailed in the Supplementary Material.

#### 3.2.2 Eosinophilic Asthma

In the subgroup with eosinophils ≥ 400 cells/μL, only the study by Busse et al. [20] evaluated this outcome. The indirect comparison suggested more favorable results with benralizumab than with reslizumab (MD = 0.11 L; 95% CI: 0.01–0.20), with no statistically significant differences observed in the remaining comparisons (Table 4).

For individuals with eosinophils ≥ 300 cells/μL, nine studies (Busse et al. [20]; Cockle et al. [26]; Ando et al. [17]; Yan et al. [21]; Akenroye et al. [10]; Nopsopon et al. [6]; Ando et al. [9]; Bateman et al. [14]; Iftikhar et al. [22]) investigated this outcome. Yan et al. [21] observed that reslizumab was associated with greater improvement than mepolizumab after 4 weeks (MD = 0.14 L; 95% CI: 0.03–0.24), although the difference was not sustained at weeks 16 and 24. Additionally, Bateman et al. [14] reported a statistically significant improvement in percent predicted FEV_1_ with dupilumab compared with omalizumab at week 52 (MD = 6.83%; 95% CI: 2.86–10.81). Finally, Ando et al. [9] reported indirect comparisons suggesting more favorable outcomes with tezepelumab (MD = 0.10 L; 95% CI: 0.01–0.19) and dupilumab (MD = 0.12 L; 95% CI: 0.02–0.20) than with benralizumab (Table 4).

### 3.3 Asthma Symptom Control—Change in ACQ Score

#### 3.3.1 General Population With Severe Uncontrolled Asthma

Three studies (Ando et al. [9]; Nachef et al. [23]; Henriksen et al. [25]) investigated this outcome in the general population with severe uncontrolled asthma, including the comparisons summarized in Table 5. Among the main findings, Ando et al. [9] reported more favorable ACQ results with mepolizumab than with dupilumab, with a statistically significant mean difference (MD = −0.24; 95% CI: −0.42 to −0.05).

**Table 5:** Statistically significant indirect comparisons of biologic therapies for asthma symptom control (ACQ), quality of life (AQLQ), and safety outcomes.

Asthma symptom control (ACQ)					
General population					
Comparison	No. of studies performing the comparison	No. of studies with significant results	Result (MD, 95% CI)	Favored drug	
Mepolizumab versus Dupilumab	1	1	Ando et al. 2022 [9]: MD = −0.24 (− 0.42 to −0.05)	Mepolizumab	

**Eosinophilic asthma**					
**Eosinophil threshold**	**Comparison**	**No. of studies performing the comparison**	**No. of studies with significant results**	**Result (MD, 95% CI)**	**Favored drug**
≥400 cells/μL	Mepolizumab versus Benralizumab	1	1	Busse et al. [20]: MD = −0.36 (−0.66 to −0.05)	Mepolizumab
	Mepolizumab versus Reslizumab	1	1	Busse et al. [20]: MD = −0.39 (−0.66 to −0.12)	Mepolizumab
≥300 cells/μL	Mepolizumab versus Benralizumab	3	1	Busse et al. [20]: MD = −0.40 (−0.76 to −0.03)	Mepolizumab
≥150 cells/μL	Tezepelumab versus Benralizumab	1	1	Ando et al. [9]: MD = −0.18 (−0.35 to −0.01)	Tezepelumab
	Mepolizumab versus Benralizumab	1	1	Busse et al. [20]: MD = −0.33 (−0.54 to −0.11)	Mepolizumab

**Quality of life (AQLQ)**					
**General population**					
**Comparison**	**No. of studies performing the comparison**	**No. of studies with significant results**	**Result (MD, 95% CI)**	**Favored drug**	
Omalizumab versus Mepolizumab	1	1	Nachef et al. [23]: MD = 0.38 (0.21–0.55)	Omalizumab	

**Eosinophilic Asthma**					
**Eosinophil threshold**	**Comparison**	**No. of studies performing the comparison**	**No. of studies with significant results**	**Result (MD, 95% CI)**	**Favored drug**
**≥150 cells/μL**	Tezepelumab versus Benralizumab	1	1	Ando et al. [9]: MD = 0.20 (0.02–0.38)	Tezepelumab
					

**Serious Adverse Events (SAEs)**					
**Comparison**	**No. of studies performing the comparison**	**No. of studies with significant results**	**Result (OR 95% CI)**	**Favored drug**	
Mepolizumab versus Dupilumab	1	1	Akenroye et al. [10]: OR = 0.65 (0.41–1.00)	Mepolizumab	

*Source:* Authors; ACQ, Asthma Control Questionnaire; AQLQ, Asthma Quality of Life Questionnaire; MD, Mean Difference; OR, Odds Ratio. Three studies have assessed the ACQ in the general population: Ando et al. [9]; Nachef et al. [23]; Henriksen et al. [25]. The change in ACQ score was analyzed in eosinophilic asthma subgroups defined by peripheral eosinophil counts (≥ 400, ≥ 300, and ≥ 150 cells/μL). Negative mean differences indicate greater improvement in ACQ score (better symptom control). Studies evaluating each subgroup: ≥ 400 cells/μL: Busse et al. [20]; ≥ 300 cells/μL: Busse et al. [20]; Akenroye et al. [10]; Nopsopon et al. [6]; Ando et al. [9]; Iftikhar et al. [22]. ≥ 150 cells/μL: Busse et al. [20]; Ando et al. [9]. Four studies have assessed the AQLQ in the general population: Ando et al. [9]; Nachef et al. [23]; Ando et al. [17]; Henriksen et al. [25]. The change in AQLQ score for eosinophilic asthma was evaluated by a single study (Ando et al. [9]) in patients with eosinophil counts ≥ 150 cells/μL. The incidence of adverse events was evaluated in five studies: Ando et al. [9], Cockle et al. [26], Ando et al. [17], Yan et al. [21], and Akenroye et al. [10]. Comparisons without statistically significant differences are further detailed in the Material S1.

#### 3.3.2 Eosinophilic Asthma

This outcome was analyzed across different subgroups of patients with eosinophilic asthma, suggesting distinct efficacy patterns among the evaluated biologics, as detailed in Table 5.

**Eosinophils** ≥ **400 cells/μL**: In this subgroup, Busse et al. [20] (2019) reported indirect comparisons suggesting more favorable outcomes with mepolizumab than with benralizumab (MD = −0.36; 95% CI: −0.66 to −0.05) and reslizumab (MD = −0.39; 95% CI: −0.66 to −0.12).

**Eosinophils** ≥ **300 cells/μL**: Five studies (Busse et al. [20]; Akenroye et al. [10]; Nopsopon et al. [6]; Ando et al. [9]; Iftikhar et al. [22]) evaluated this outcome. In Busse et al. [20], indirect comparisons suggested more favorable outcomes with mepolizumab than with benralizumab (MD = −0.40; 95% CI: −0.76 to −0.03).

**Eosinophils** ≥ **150 cells/μL**: This subgroup was evaluated by Busse et al. [20] and Ando et al. [9]. In Ando et al. [9], indirect comparisons suggested more favorable outcomes with tezepelumab than with benralizumab (MD = −0.18; 95% CI: −0.35 to −0.01), while in Busse et al. [20], indirect comparisons suggested more favorable outcomes with mepolizumab than with benralizumab (MD = −0.33; 95% CI: −0.54 to −0.11) (Table 5).

### 3.4 Asthma‐Related Quality of Life—Change in AQLQ Score

#### 3.4.1 General Population With Severe Uncontrolled Asthma

Four studies (Ando et al. [9]; Nachef et al. [23]; Ando et al. [17]; Henriksen et al. [25]) evaluated this outcome. According to Nachef et al. [23] (2018), indirect comparisons suggested more favorable AQLQ results with omalizumab than with mepolizumab (MD = 0.38; 95% CI: 0.21–0.55) (Table 5).

#### 3.4.2 Eosinophilic Asthma

**Eosinophils** ≥ **150 cells/μL:** In the eosinophilic subgroup, Ando et al. [9] compared tezepelumab and benralizumab, reporting more favorable outcomes with tezepelumab (MD = 0.199; 95% CI: 0.018–0.382) (Table 5).

### 3.5 Safety Outcomes

Five studies (Ando et al. [9]; Cockle et al. [26]; Ando et al. [17]; Yan et al. [21]; Akenroye et al. [10]) evaluated safety outcomes. Indirect comparisons by Akenroye et al. [10] suggested lower odds of serious adverse events with mepolizumab than with dupilumab (OR = 0.65; 95% CI: 0.41–1.00) (Table 5).

### 3.6 Reduction in Oral Corticosteroid Dose

Two studies (Phinyo et al. [11]; Bourdin et al. [16]) assessed this outcome, including multiple comparisons among biologic agents. Phinyo et al. [11] reported indirect comparisons suggesting more favorable outcomes with benralizumab and dupilumab in the following comparisons: benralizumab versus tezepelumab: OR = 3.20 (95% CI: 1.34‐7.60) (4 weeks), OR = 3.22 (95% CI: 1.35‐7.68) (8 weeks); benralizumab versus reslizumab: OR = 3.33 (95% CI: 1.45‐7.65) (4 weeks), OR = 3.35 (95% CI: 1.45‐7.72) (8 weeks); dupilumab versus tezepelumab: OR = 2.54 (95% CI: 1.12‐5.73); and dupilumab versus reslizumab: OR = 2.64 (95% CI: 1.21‐5.75).

### 3.7 Eosinophil Count

Yan et al. [21] compared reslizumab and mepolizumab, observing a statistically significant reduction in eosinophil count with reslizumab across multiple time points: 4 weeks: MD = −165.23 (95% CI: −255.97 to −73.92); 16 weeks: MD = −117.03 (95% CI: −197.01 to −37.11); and 24 weeks: MD = −202.17 (95% CI: −312.23 to −94.86).

### 3.8 Methodological Quality Assessment—AMSTAR

The methodological quality of the included systematic reviews was assessed using the AMSTAR 2 tool. Among the 17 studies analyzed, five were rated as having low methodological quality, while 12 were classified as critically low.

Reviews rated as low quality failed to meet at least one critical AMSTAR 2 domain. These studies exhibited less severe limitations, such as lack of justification for study exclusions and incomplete assessment of publication bias. However, they typically registered a review protocol and employed reasonably comprehensive search strategies.

In contrast, studies rated as critically low violated two or more critical domains. The most common methodological flaws included the lack of a registered protocol, inadequate or incomplete search strategies, lack of justification for excluded studies, insufficient assessment of publication bias, and lack of duplicate processes for study selection and data extraction.

Overall, the most frequent contributors to methodological downgrading were lack of protocol registration, insufficient justification for exclusions, suboptimal publication bias analysis, and lack of duplicate review procedures. These limitations substantially compromise the reliability of the findings and underscore the need for cautious interpretation of the results (Table 6). The detailed results of this assessment are presented in the Material S1.

**Table 6:** Summary of methodological quality assessment of the included systematic reviews using the AMSTAR 2 tool.

Study	Methodological quality	Rationale for classification
Phinyo et al. [11]	Low	The protocol was registered, but with inconsistencies in the publication bias analysis and insufficient justification for study exclusions.
Akenroye et al. [10]	Low	The protocol was registered, but it lacked adequate justification for exclusions and included only a partial analysis of publication bias.
Nopsopon et al. [6]	Low	The protocol was registered, but the search of grey literature was incomplete, and the justification for exclusions was insufficient.
Ando et al. [9]	Critically low	No registered protocol, incomplete search strategies, and a lack of adequate justification for exclusions.
Bateman et al. [14]	Critically low	No registered protocol, incomplete search strategies, failures in duplicate data extraction, and no formal analysis of publication bias.
Menzies‐Gow et al. [15]	Critically low	No registered protocol, flawed search strategies, data extraction was not performed in duplicate, and no formal analysis of publication bias.
Bourdin et al. [16]	Critically low	No registered protocol, inadequate search strategies, and a lack of detailed justification for exclusions.
Ando et al. [17]	Low	The protocol was registered, but search strategies were incomplete and lacked detailed justification for exclusions.
Ramonell & Iftikhar. [18]	Critically low	No registered protocol, flawed search strategies and bias analysis, and a lack of adequate justification for exclusions.
Edris et al. [19]	Critically low	No registered protocol, insufficient search strategies, a lack of detailed justification for exclusions, and an inadequate analysis of publication bias.
Busse et al. [20]	Critically low	No registered protocol, no duplication in data extraction, a lack of adequate justification for exclusions, and an inadequate analysis of publication bias.
Yan et al. [21]	Low	The protocol was registered, but search strategies were incomplete, and justifications for exclusions were partial.
Iftikhar et al. [22]	Critically low	No registered protocol, study selection, and data extraction were not performed in duplicate, and there was an absence of detailed justification for exclusions.
Nachef et al. [23]	Critically low	No registered protocol, incomplete search strategies, and a lack of detailed justification for exclusions.
Bourdin et al. [24]	Critically low	No registered protocol, limited search strategy, study selection, and data extraction were not performed in duplicate, and there was an absence of detailed justification for exclusions.
Henriksen et al. [25]	Critically low	No registered protocol, incomplete search strategies, and a lack of detailed justification for exclusions.
Cockle et al. [26]	Critically low	No registered protocol, incomplete search strategies, a lack of detailed justification for exclusions, and no formal analysis of publication bias.

*Source:* Authors.

## 4 Discussion

Comparative studies of pharmacological treatments play a pivotal role in clinical decision‐making, supporting evidence‐based choices and guiding the selection of the most effective and safest options tailored to patient profiles [27]. In this context, the present overview of systematic reviews provides a comprehensive synthesis of the available evidence on the efficacy and safety of biologic therapies for uncontrolled asthma.

The analysis of biologic agents for asthma treatment identified potential differences in efficacy, particularly regarding the annual exacerbation rate (AER). In the general population, dupilumab (compared with benralizumab, mepolizumab, and reslizumab) and tezepelumab (compared with benralizumab) were associated with more favorable outcomes in indirect comparisons, whereas benralizumab (compared with mepolizumab, tezepelumab, and reslizumab) was associated with more favorable outcomes among patients dependent on oral corticosteroids. The more favorable outcomes observed with tezepelumab and dupilumab may be related, at least in part, to their broader spectrum of action, as these agents target multiple inflammatory pathways, unlike other biologics that specifically inhibit the IL‐5 pathway [28, 29, 30].

Asthma exacerbations remain among the leading causes of morbidity and mortality, substantially increasing healthcare utilization and treatment costs [31]. In this context, the AER represents a clinically relevant outcome, directly reflecting disease severity and its impact on patients' quality of life. These findings support the use of the AER as a key indicator of therapeutic response and justify the evaluation of biologics within specific patient subgroups, particularly according to baseline eosinophil counts.

In patients with eosinophilic asthma, the efficacy of biologic therapies varied according to baseline eosinophil counts. For counts ≥ 400 cells/μL, indirect comparisons suggested more favorable outcomes with mepolizumab than with reslizumab and benralizumab. At ≥ 300 cells/μL, indirect evidence suggested more favorable outcomes with mepolizumab than with benralizumab, while dupilumab was also associated with more favorable outcomes than benralizumab; indirect evidence suggested more favorable outcomes with reslizumab than with mepolizumab, and indirect comparisons also suggested more favorable outcomes with tezepelumab than with benralizumab. Among patients with ≥ 150 cells/μL, dupilumab, mepolizumab, and tezepelumab were associated with more favorable outcomes than benralizumab, with tezepelumab also showing more favorable outcomes than omalizumab. In individuals with < 150 cells/μL, indirect comparisons suggested more favorable outcomes with tezepelumab than with dupilumab. The larger number of studies available for the ≥ 150 and ≥ 300 cells/μL subgroups contributed to greater consistency of findings across studies.

Given the well‐recognized importance of baseline eosinophil counts in tailoring therapy for severe asthma [32], these findings are clinically relevant. Accordingly, selecting a biologic agent according to the patient's clinical and inflammatory profile, as suggested by the available evidence, may contribute to clinical benefit and more efficient use of healthcare resources. Beyond exacerbation reduction, another clinically relevant outcome is improvement in lung function (FEV_1_). Regarding pulmonary function, dupilumab appeared to be associated with greater improvements than benralizumab and reslizumab in individuals with uncontrolled severe asthma. In subgroup analyses of eosinophilic asthma, additional differences were observed: indirect comparisons suggested more favorable outcomes with benralizumab than with reslizumab in patients with baseline eosinophil counts ≥ 400 cells/μL; dupilumab was associated with greater improvements than benralizumab and omalizumab; and tezepelumab was associated with more favorable outcomes than benralizumab in the moderate eosinophilia range (≥ 300 cells/μL). Furthermore, indirect evidence suggested more favorable outcomes with reslizumab than with mepolizumab at the same threshold.

The more favorable outcomes observed with dupilumab may be related to its mechanism of action, which blocks interleukin‐4 and interleukin‐13 signaling, thereby reducing airway inflammation and promoting improvement in lung function [33, 34, 35]. The more favorable outcomes observed with reslizumab compared with mepolizumab in indirect comparisons are consistent with findings from previous systematic reviews that compared these biologics with placebo [36, 37].

Regarding clinical control and the reduction of symptom frequency and intensity (ACQ), indirect evidence suggested more favorable clinical outcomes with mepolizumab than with dupilumab in the general population and with benralizumab and reslizumab in patients with baseline eosinophil counts of ≥ 150, ≥ 300, and ≥ 400 cells/μL. Similarly, indirect comparisons suggested more favorable outcomes with tezepelumab (in patients with counts ≥ 150 cells/μL) than with benralizumab. These effects may be partly explained by modulation of key immunologic pathways, including attenuation of eosinophilic inflammation, resulting in symptom relief and reduced need for rescue medication [38]. Moreover, the more favorable outcomes associated with mepolizumab in this overview are consistent with the findings of other studies [39, 40, 41].

Although benefits may be more pronounced in patients with elevated eosinophil counts, heterogeneous baseline levels and inter‐study variability underscore the need for individualized therapeutic strategies. Incorporating inflammatory biomarkers, disease severity, and patient profiles may help optimize clinical outcomes and improve quality of life. Additionally, it is important to consider the impact of biologic therapies on patients' quality of life. Improvements in AQLQ were more evident among patients treated with omalizumab (general population) than among those treated with mepolizumab, and among those receiving tezepelumab (baseline eosinophil counts ≥ 150 cells/μL) than among those receiving benralizumab.

These effects may be associated with reduced allergen hypersensitivity, leading to a lower symptom burden and decreased dependence on pharmacological intervention [42, 43]. However, each of these statistically significant differences was observed in a single comparison, and the included studies displayed high heterogeneity, which limits the generalizability of the findings. Therefore, further well‐designed, direct comparative studies with larger sample sizes and methodological standardization are needed to confirm these results and provide more robust evidence on the relative impact of biologic therapies on the quality of life of patients with uncontrolled severe asthma.

Beyond efficacy and symptom control, safety remains a critical consideration in the clinical use of biologic therapies. In this overview of systematic reviews, mepolizumab was associated with lower odds of serious adverse events compared with dupilumab in the general asthma population. However, this finding was derived from indirect comparisons, and only one of the five studies evaluating safety outcomes reported a statistically significant difference between treatments. This potentially favorable safety profile may be related to mepolizumab's selective mechanism of action, although this observation should be interpreted with caution given the limited evidence available [44, 45].

Regarding other clinical outcomes, indirect comparisons suggested more favorable outcomes with benralizumab than with reslizumab and tezepelumab in reducing the dose of oral corticosteroids, while dupilumab was also associated with more favorable outcomes than reslizumab and tezepelumab. Prolonged use of oral corticosteroids is associated with several adverse effects, including increased morbidity and mortality in patients with severe asthma [11]. In this context, biologic therapies capable of reducing the need for oral corticosteroids may offer important additional clinical benefits. Taken together, these findings support the potential benefits of biologic therapies not only on clinical outcomes but also on inflammatory markers, such as eosinophil counts, which showed a consistent reduction with reslizumab over 24 weeks [1, 42].

This overview of systematic reviews reinforces the importance of selecting biologic agents according to patients' clinical and phenotypic characteristics to optimize asthma management and reduce the need for oral corticosteroids, thereby minimizing their adverse effects. By synthesizing evidence from systematic reviews that used meta‐analytical and indirect‐comparison methods, this study provides a comprehensive synthesis of the currently available evidence on the efficacy and safety of biologic therapies, contributing to more informed and individualized clinical decision‐making. Furthermore, by integrating results from different clinical and methodological contexts, this overview helps identify patterns of therapeutic response and offers valuable insights for the personalized management of severe asthma.

An important aspect to consider when interpreting these findings is the methodological quality of the included systematic reviews. According to the AMSTAR 2 assessment, most reviews were classified as having critically low methodological quality, mainly due to the lack of protocol registration, incomplete search strategies, insufficient justification for study exclusions, and limitations in publication bias assessment. Consequently, although some biologics appeared to be associated with favorable outcomes in specific clinical settings, these comparative findings should be interpreted cautiously, particularly against the lack of direct head‐to‐head randomized clinical trials and given the limited certainty of the available evidence.

Several limitations should be acknowledged, which include heterogeneity among the included studies, variation in inclusion criteria, and potential overlap of primary studies across the systematic reviews and network meta‐analyses included in this overview. A formal quantitative assessment of overlap, such as the corrected covered area (CCA), was not performed. Since some reviews synthesized evidence from similar clinical trials, certain primary studies may have contributed more than once to the overall body of evidence, potentially increasing the influence of specific trials on the interpretation of comparative efficacy and safety outcomes. In addition, some trials were sponsored by the pharmaceutical industry, which may represent a potential source of sponsorship bias. Therefore, these findings should be interpreted with caution, particularly because statistically significant differences between biologic agents were observed only in selected comparisons. The evidence underscores the need for further independent research with greater methodological standardization and stricter management of overlapping evidence to provide more precise guidance for clinical practice.

Nevertheless, the findings offer a useful and up‐to‐date perspective on the role of biologic therapies in severe asthma, representing a meaningful contribution to clinical practice, the development of therapeutic guidelines, and future research in the field.

## 5 Conclusions

This overview of systematic reviews suggests that biologic therapies may play an important role in the management of uncontrolled asthma, particularly among patients with different eosinophilic profiles or corticosteroid dependence. Dupilumab, benralizumab, mepolizumab, and tezepelumab were reported to be associated with reduced exacerbation rates in several indirect comparisons; dupilumab and tezepelumab showed favorable results for lung function; and mepolizumab and tezepelumab were associated with improved symptom control in specific patient subgroups. In addition, omalizumab and tezepelumab demonstrated potential benefits in improving patients' quality of life.

Regarding safety, one indirect comparison suggested a potentially more favorable serious adverse‐event profile for mepolizumab than for dupilumab, although this finding should be interpreted cautiously. Collectively, these findings support the role of biologics as personalized treatment strategies that may contribute to improved clinical outcomes, reduced corticosteroid requirements, and more targeted management of moderate‐to‐severe asthma.

However, the comparative findings presented in this overview should be interpreted with caution due to methodological heterogeneity and the predominance of systematic reviews classified as having critically low methodological quality according to the AMSTAR 2 assessment. Therefore, although potentially important differences among biologics were identified in specific clinical settings, the current evidence remains limited for establishing definitive comparative rankings among therapies.

Nevertheless, this synthesis provides a comprehensive overview of the currently available evidence, highlighting the importance of individualized treatment decisions and the selection of the most appropriate biologic agent according to patient characteristics. Furthermore, it reinforces the need for future independent, methodologically rigorous, and standardized studies to strengthen current recommendations and support more precise therapeutic decision‐making.

## Author Contributions

**Ronaldo José Faria:** conceptualization, investigation, writing – original draft, writing – review and editing, methodology, validation, visualization, data curation, formal analysis. **Patrícia Silva Bazoni:** conceptualization, investigation, writing – original draft, writing – review and editing, software, formal analysis, validation, methodology, data curation, visualization. **Jéssica Barreto Ribeiro dos Santos:** conceptualization, investigation, writing – original draft, writing – review and editing, validation, methodology, software, formal analysis, data curation, visualization. **Erica Tatiane da Silva:** conceptualization, investigation, formal analysis, software, data curation, validation, methodology, writing – original draft, visualization. **Michael Ruberson Ribeiro da Silva:** supervision, data curation, validation, methodology, writing – review and editing, writing – original draft, conceptualization, investigation.

## Funding

The authors have nothing to report.

## Conflicts of Interest

The authors declare no conflicts of interest.

## Supporting information

Supporting File 1

## Data Availability

The data supporting the conclusions of this study are available in the Supplementary Material to this article.
