# Supplementary material for: Efficacy and Safety of Biologic Therapies for Uncontrolled Asthma: An Overview of Systematic Reviews
Source: Pediatr Pulmonol. 2026 Jul 7;61(7):e71727. doi: 10.1002/ppul.71727 (PMC13338779; doi:10.1002/ppul.71727)
Supplement: Supplementary file 1 — Supporting File 1 [file PPUL-61-0-s001.pdf]

EFFICACY AND SAFETY OF BIOLOGIC THERAPIES FOR UNCONTROLLED ASTHMA: AN OVERVIEW OF SYSTEMATIC REVIEWS

SUPPLEMENTAL MATERIAL

1 EXCLUDED STUDIES

The table below lists the studies that were excluded from the review, along with the specific reasons for their exclusion.

Study	Reason for exclusion
Agache et al. (2020)	Did not perform meta-analysis or indirect comparison
Akdis et al. (2020)	Did not perform meta-analysis or indirect comparison
Alam et al. (2023)	Did not perform meta-analysis or indirect comparison
Alhossan et al. (2017)	Did not perform meta-analysis or indirect comparison
Ayyagari et al. (2016)	Ineligible publication type
Bacharier et al. (2018)	Ineligible publication type
Bassani et al. (2019)	Did not perform meta-analysis or indirect comparison
Bhavanasai et al. (2023)	Ineligible publication type
Belhadi et al. (2015)	Ineligible publication type
Bergrath et al. (2014)	Ineligible publication type
Benson et al. (2022)	Did not perform meta-analysis or indirect comparison
Boulton & Shen (2022)	Did not perform meta-analysis or indirect comparison
Bousqueta et al. (2007)	Did not perform meta-analysis or indirect comparison
Cadth (2018)	Ineligible publication type
Calzetta et al. (2021)	Did not perform meta-analysis or indirect comparison
Caminati et al. (2015)	Did not perform meta-analysis or indirect comparison
Casale et al. (2019)	Did not perform meta-analysis or indirect comparison
Charles et al. (2022)	Did not perform meta-analysis or indirect comparison
Chen et al. (2019)	Did not perform meta-analysis or indirect comparison
Chen et al. (2023)	Did not perform meta-analysis or indirect comparison
Corren et al. (2015)	Ineligible publication type
Crossingham et al. (2022)	Did not meet study design eligibility criteria
Edris; Lahousse (2021)	Did not perform meta-analysis or indirect comparison
Emma et al. (2018)	Did not perform meta-analysis or indirect comparison
Fainardi et al. (2016)	Did not perform meta-analysis or indirect comparison
Faulkner et al. (2021)	Did not perform meta-analysis or indirect comparison
Henriksen et al. (2020)	Did not perform meta-analysis or indirect comparison
Israel et al. (2022)	Did not perform meta-analysis or indirect comparison
Kodaka et al. (2023)	Ineligible publication type
Korn et al. (2023)	Did not perform meta-analysis or indirect comparison
Kyriakopoulos et al. (2024)	Did not perform meta-analysis or indirect comparison
Lai et al. (2015)	Did not perform meta-analysis or indirect comparison
Leung et al. (2017)	Did not perform meta-analysis or indirect comparison

Li et al. (2021)	Did not perform meta-analysis or indirect comparison
Liao et al. (2024)	Did not perform meta-analysis or indirect comparison
MacDonald et al. (2019)	Did not perform meta-analysis or indirect comparison
Manka & Wechsler (2018)	Did not meet study design eligibility criteria
Martin et al. (2022)	Did not perform meta-analysis or indirect comparison
Menzela et al. (2019)	Did not perform meta-analysis or indirect comparison
Meteran et al. (2017)	Ineligible publication type
Miranda (2020)	Did not perform meta-analysis or indirect comparison
Morjaria et al. (2019)	Did not perform meta-analysis or indirect comparison
Nissen et al. (2019)	Ineligible publication type
Norman et al. (2013)	Did not meet study design eligibility criteria
Nowak (2005)	Did not perform meta-analysis or indirect comparison
Ortega et al. (2023)	Ineligible publication type
Özdemir & Bavbek (2017)	Ineligible publication type
Patrick et al. (2021)	Did not perform meta-analysis or indirect comparison
Pavord et al. (2021)	Ineligible publication type
Plaza et al. (2023)	Did not perform meta-analysis or indirect comparison
Robbins et al. (2021)	Ineligible publication type
Sharma et al. (2016)	Ineligible publication type
Spahn et al. (2023)	Did not perform meta-analysis or indirect comparison
Taleb & Badgett (2021)	Ineligible publication type
Torvinen et al. (2015)	Ineligible publication type
Tran et al. (2019)	Ineligible publication type
Virchow et al. (2020)	Ineligible publication type
Wang et al. (2016)	Did not perform meta-analysis or indirect comparison
Yang et al. (2018)	Did not perform meta-analysis or indirect comparison

2 SEARCH STRATEGY

A comprehensive literature search was conducted across five databases to identify studies related to the efficacy and safety of biologic therapies for asthma. The search strategy included both controlled vocabulary and free-text terms covering asthma-related conditions and the target biologic agents. Filters were applied to retrieve systematic reviews and meta-analyses.

2.1 Embase – 821 results

('asthma'/exp OR 'asthma' OR 'asthma bronchiale' OR 'asthma pulmonale' OR 'asthma, bronchial' OR 'asthmatic' OR 'asthmatic subject' OR 'bronchial asthma' OR 'bronchus asthma' OR 'chronic asthma' OR 'lung allergy') AND ('benralizumab'/exp OR 'benralizumab' OR 'biw 8405' OR 'biw8405' OR 'fasenra' OR 'khk 4563' OR 'khk4563' OR 'medi 563' OR 'medi563' OR 'dupilumab'/exp OR 'bat 2406' OR 'bat2406' OR 'dupilumab' OR 'dupixent' OR 'regn 668' OR 'regn668' OR 'sar 231893' OR 'sar231893' OR 'mepolizumab'/exp OR 'bat 2606' OR 'bat2606' OR 'bosatria' OR 'mepolizumab' OR 'nucala' OR 'sb 240563' OR 'sb-240563' OR 'sb240563' OR 'omalizumab'/exp OR 'fb 317' OR 'fb317' OR 'gbr 310' OR 'gbr310' OR 'hu 901' OR 'hu901' OR 'monoclonal antibody e 25' OR 'monoclonal antibody e25' OR 'olizumab' OR 'omalizumab' OR 'rg 3648' OR 'rg3648' OR 'rhumab 25' OR 'rhumab e25' OR 'sti 004' OR 'sti004' OR 'syn 008' OR 'syn008' OR 'xolair' OR 'reslizumab'/exp OR 'cep 38072' OR 'cep38072' OR 'cinqaero' OR 'cinqair' OR 'dcp 835' OR 'dcp835' OR 'reslizumab' OR 'sch 55700' OR 'sch55700' OR 'tezepelumab'/exp OR 'amg 157' OR 'amg157' OR 'medi 9929' OR 'medi9929' OR 'tezepelumab' OR 'tezepelumab ekko' OR 'tezepelumab-ekko' OR 'tezspire') AND ('meta analysis (topic)'/exp OR 'meta analysis'/exp OR ((meta NEXT/1 analy*):ab,ti) OR metaanaly*:ab,ti OR 'systematic review (topic)'/exp OR 'systematic review'/exp OR ((systematic NEXT/1 review*):ab,ti) OR ((systematic NEXT/1 overview*):ab,ti) OR cancerlit:ab,ti OR cochrane:ab,ti OR embase:ab,ti OR psychlit:ab,ti OR psyclit:ab,ti OR psychinfo:ab,ti OR psycinfo:ab,ti OR cinahl:ab,ti OR cinhal:ab,ti OR 'science citation index':ab,ti OR bids:ab,ti OR ((reference NEXT/1 list*):ab,ti) OR bibliograph*:ab,ti OR 'hand search*':ab,ti OR ((manual NEXT/1 search*):ab,ti) OR 'relevant journals':ab,ti OR (('data extraction':ab,ti OR 'selection criteria':ab,ti) AND review/it)) NOT (letter/it OR editorial/it OR ('animal'/exp NOT ('animal'/exp AND 'human'/exp)))

2.2 Medline via PubMed – 426 results

(("Asthma"[MeSH Terms] OR "Asthma"[Text Word] OR "Asthmas"[Text Word] OR "asthma bronchial"[Text Word] OR "bronchial asthma"[Text Word]) AND ("benralizumab"[Supplementary Concept] OR "benralizumab"[Text Word] OR "MEDI-563"[Text Word] OR "MEDI563"[Text Word] OR "Fasenra"[Text Word] OR "BIW-8405"[Text Word]) OR ("dupilumab"[Supplementary Concept] OR "dupilumab"[Text Word] OR "SAR231893"[Text Word] OR "SAR-231893"[Text Word] OR "REGN-668"[Text Word] OR "REGN668"[Text Word] OR "Dupixent"[Text Word]) OR ("mepolizumab"[Supplementary Concept] OR "mepolizumab"[Text Word] OR "SB-240563"[Text Word] OR "SB240563"[Text Word] OR "Nucala"[Text Word] OR "Bosatria"[Text Word]) OR ("Omalizumab"[MeSH Terms] OR "Omalizumab"[Text Word] OR "Xolair"[Text Word]) OR ("reslizumab"[Supplementary Concept] OR "reslizumab"[Text Word] OR "Cinqair"[Text Word] OR "SCH-55700"[Text Word] OR "SCH55700"[Text Word] OR "DCP-835"[Text Word] OR "DCP835"[Text Word] OR "CEP-38072"[Text Word] OR "CEP38072"[Text Word])OR ("tezepelumab"[Supplementary Concept] OR "tezepelumab"[Text Word] OR "MEDI-9929"[Text Word] OR "MEDI9929"[Text Word] OR "AMG-157"[Text Word] OR "tezspire"[Text Word] OR "tezepelumab-ekko"[Text Word])) AND (("systematic review"[Publication Type] OR "systematic reviews as topic"[MeSH Terms] OR "systematic review"[Text Word]) OR ("meta-analysis"[Publication Type] OR "meta-analysis as topic"[MeSH Terms] OR "meta-analys*"[Text Word] OR meta analys* OR metaanalys*))

2.3 LILACS – 3 results

("Asthma" OR "Asthmas" OR "asthma bronchial" OR "bronchial asthma") AND (("benralizumab" OR "MEDI-563" OR "Fasenra" OR "BIW-8405") OR ("dupilumab" OR "SAR231893" OR "SAR-231893" OR "REGN668" OR "REGN-668" OR "Dupixent") OR ("mepolizumab" OR "SB-240563" OR "SB240563" OR "Nucala" OR "Bosatria") OR ("Omalizumab" OR "Xolair") OR ("reslizumab" OR "Cinqair" OR "SCH-55700" OR "SCH55700" OR "DCP-835" OR "DCP835" OR "CEP-38072" OR "CEP38072") OR ("tezepelumab" OR "MEDI-9929" OR "MEDI9929" OR "AMG-157" OR "tezspire" OR "tezepelumab-ekko")) AND ((Systematic review$) OR (Meta- analy$) OR (Meta analy$) OR (Metaanaly$)))

2.4 Cochrane Central – 22 results

ID Search Hits
#1 MeSH descriptor: [Asthma] explode all trees 15034
#2 "Asthma" OR "Asthmas" OR "asthma bronchial" OR "bronchial asthma" 38768
#3 #1 OR #2 38777
#4 "benralizumab" OR "MEDI-563" OR "MEDI563" OR "Fasenra" OR "BIW-8405" 311
#5 "dupilumab" OR "SAR231893" OR "SAR-231893" OR "REGN668" OR "REGN-668" OR "Dupixent" 1222
#6 "mepolizumab" OR "SB-240563" OR "SB240563" OR "Nucala" OR "Bosatria" 464
#7 "Omalizumab" OR "Xolair" 1145
#8 "reslizumab" OR "Cinqair" OR "SCH-55700" OR "SCH55700" OR "DCP-835" OR "DCP835" OR "CEP-38072" OR "CEP38072" 145
#9 "tezepelumab" OR "MEDI-9929" OR "MEDI9929" OR "AMG-157" OR "tezspire" OR "tezepelumab-ekko" 208
#10 #4 OR #5 OR #6 OR #7 OR #8 OR #9 3288
#11 #3 AND #10

2.5 Web of Science – 289 results

((ALL=(("Asthma" OR "Asthmas" OR "asthma bronchial" OR "bronchial asthma"))) AND ALL=(("benralizumab" OR "MEDI-563" OR "Fasenra" OR "BIW-8405") OR ("dupilumab" OR "SAR231893" OR "SAR-231893" OR "REGN668" OR "REGN-668" OR "Dupixent") OR ("mepolizumab" OR "SB-240563" OR "SB240563" OR "Nucala" OR "Bosatria") OR ("Omalizumab" OR "Xolair") OR ("reslizumab" OR "Cinqair" OR "SCH-55700" OR "SCH55700" OR "DCP-835" OR "DCP835" OR "CEP-38072" OR "CEP38072") OR ("tezepelumab" OR "MEDI-9929" OR "MEDI9929" OR "AMG-157" OR "tezspire" OR "tezepelumab-ekko")))) AND ALL=(("Systematic review" OR "Meta-analys*" OR "Meta analys*" OR "Metaanalys*"))

3. RESULTS BY OUTCOME

3.1 Annual Exacerbation Rate

3.1.1 Severe Uncontrolled Asthma (General Population)

Ando et al. (2022)	Tezepelumab vs. Mepolizumab: RR = 0.991 (95% CrI: 0.751–1.310)<br>Tezepelumab vs. Benralizumab: RR = 0.734 (0.588–0.916)*<br>Tezepelumab vs. Dupilumab: RR = 0.815 (0.609–1.092)<br>Dupilumab vs. Mepolizumab: RR = 1.216 (0.889–1.662)<br>Dupilumab vs. Benralizumab: RR = 0.901 (0.692–1.172)<br>Benralizumab vs. Mepolizumab: RR = 1.350 (1.052–1.732)*<br><br>**SUCRA ranking:** Tezepelumab: 86.0%, Mepolizumab: 83.8%, Benralizumab: 30.8%, Dupilumab: 49.4%
Bateman et al. (2022)	Dupilumab 200 mg vs. Benralizumab (Q8W): RR = 0.46 (0.32–0.66)<br>Dupilumab 300 mg vs. Benralizumab (Q8W): RR = 0.45 (0.30–0.65)<br>Dupilumab 200/300 mg vs. Benralizumab (Q8W): RR = 0.46 (0.32–0.67)*<br>Dupilumab 200 mg vs. Mepolizumab: RR = 0.68 (0.50–0.93)<br>Dupilumab 300 mg vs. Mepolizumab: RR = 0.79 (0.58–1.09)<br>Dupilumab 200/300 mg vs. Mepolizumab: RR = 0.72 (0.57–0.92)*<br>Dupilumab 200 mg vs. Reslizumab (Q4W): RR = 0.58 (0.43–0.80)<br>Dupilumab 300 mg vs. Reslizumab (Q4W): RR = 0.45 (0.13–1.58)<br>Dupilumab 200/300 mg vs. Reslizumab (Q4W): RR = 0.62 (0.48–0.79)*
Menzies-Gow et al. (2022)	Tezepelumab vs. Dupilumab: RR = 0.84 (0.45–1.56) for both 200 mg and 300 mg<br>Tezepelumab vs. Benralizumab: RR = 0.63 (0.35–1.09)<br>Tezepelumab vs. Mepolizumab: RR = 0.82 (0.43–1.50)<br>Tezepelumab vs. Reslizumab: RR = 0.82 (0.43–1.49)<br>Tezepelumab vs. Omalizumab: RR = 0.60 (0.35–1.01)<br>Dupilumab 200mg vs. Tezepelumab: RR = 1.19 (0.64–2.22)<br>Dupilumab 200mg vs. Dupilumab 300mg: RR = 1.00 (0.58–1.71)<br>Dupilumab 200mg vs. Benralizumab: RR = 0.75 (0.43–1.28)<br>Dupilumab 200mg vs. Mepolizumab: RR = 0.98 (0.52–1.77)<br>Dupilumab 200mg vs. Reslizumab: RR = 0.97 (0.52–1.76)<br>Dupilumab 200mg vs. Omalizumab: RR = 0.71 (0.42–1.18)<br>Dupilumab 300mg vs. Tezepelumab: RR = 1.19 (0.64–2.22)<br>Dupilumab 300mg vs. Dupilumab 200mg: RR = 1.00 (0.58–1.71)<br>Dupilumab 300mg vs. Benralizumab: RR = 0.75 (0.43–1.28)<br>Dupilumab 300mg vs. Mepolizumab: RR = 0.98 (0.52–1.77)<br>Dupilumab 300mg vs. Reslizumab: RR = 0.97 (0.52–1.76)<br>Dupilumab 300mg vs. Omalizumab: RR = 0.71 (0.42–1.18)<br>Benralizumab vs. Tezepelumab: RR = 1.59 (0.92–2.86)<br>Benralizumab vs. Dupilumab 200mg: RR = 1.33 (0.78–2.33)<br>Benralizumab vs. Dupilumab 300mg: RR = 1.33 (0.78–2.33)<br>Benralizumab vs. Mepolizumab: RR = 1.32 (0.76–2.27)<br>Benralizumab vs. Reslizumab: RR = 1.30 (0.76–2.22)<br>Benralizumab vs. Omalizumab: RR = 0.96 (0.61–1.48)<br>Mepolizumab vs. Tezepelumab: RR = 1.22 (0.67–2.33)<br>Mepolizumab vs. Dupilumab 200mg: RR = 1.02 (0.56–1.92)<br>Mepolizumab vs. Dupilumab 300mg: RR = 1.02 (0.56–1.92)<br>Mepolizumab vs. Benralizumab: RR = 0.76 (0.44–1.32)<br>Mepolizumab vs. Reslizumab: RR = 0.99 (0.54–1.81)<br>Mepolizumab vs. Omalizumab: RR = 0.73 (0.43–1.21)<br>Reslizumab vs. Tezepelumab: RR = 1.22 (0.67–2.33)<br>Reslizumab vs. Dupilumab 200mg: RR = 1.03 (0.57–1.92)<br>Reslizumab vs. Dupilumab 300mg: RR = 1.03 (0.57–1.92)<br>Reslizumab vs. Benralizumab: RR = 0.77 (0.45–1.32)

	Reslizumab vs. Mepolizumab: RR = 1.01 (0.55–1.85)<br>Reslizumab vs. Omalizumab: RR = 0.74 (0.44–1.22)<br>Omalizumab vs. Tezepelumab: RR= 1,67 (0,99–2,86)<br>Omalizumab vs. Dupilumab 200mg: RR= 1,41 (0,85–2,38)<br>Omalizumab vs. Dupilumab 300mg: RR= 1,39 (0,85–2,38)<br>Omalizumab vs. Benralizumab: RR= 1,04 (0,68–1,64)<br>Omalizumab vs. Mepolizumab: RR= 1,37 (0,83–2,33)<br>Omalizumab vs. Reslizumab: RR= 1,35 (0,82–2,27)<br><br>**SUCRA ranking:** Tezepelumab: 84%, Dupilumab (200/300 mg): 66%, Mepolizumab: 64%, Reslizumab: 63%, Benralizumab: 31%, Omalizumab: 26%.
Ando et al. (2020)	Dupilumab vs. Benralizumab: RR = 0.83 (0.62–1.09)
Ramonell & Iftikhar (2020)	***Log rate ratio comparisons:***<br>Dupilumab vs. Mepolizumab: −0.16 (−0.76 to 0.44)<br>Dupilumab vs. Reslizumab: −0.19 (−0.91 to 0.53)<br>Dupilumab vs. Benralizumab: −0.45 (−1.06 to 0.15)<br>Mepolizumab vs. Reslizumab: −0.03 (−0.76 to 0.69)<br>Mepolizumab vs. Benralizumab: −0.29 (−0.90 to 0.32)<br>Reslizumab vs. Benralizumab: −0.26 (−0.99 to 0.47)
Bourdin et al. (2018)	Benralizumab (Q8W) vs. Mepolizumab (no matching adjustment): RR = 1.06 (0.88–1.28); p = 0.5553<br>Benralizumab (Q8W) vs. Mepolizumab (with matching adjustment): RR = 0.94 (0.78–1.13); p = 0.5207

Source: Authors. Q4W: Every 4 weeks; Q8W: Every 8 weeks; RR: Rate Ratio; * significant.

3.1.2 Eosinophilic Asthma

Akenroye et al. (2022)	***Eosinophils ≥ 300 cells/µL:***<br>Dupilumab vs. Benralizumab: RR = 0.66 (0.47–0.94)*<br>Mepolizumab vs. Benralizumab: RR = 0.75 (0.60–0.95)*<br>Mepolizumab vs. Dupilumab: RR = 1.10 (0.74–1.70)<br><br>***Eosinophils 150–299 cells/µL:***<br>Dupilumab vs. Benralizumab: RR = 0.97 (0.59–1.60)<br>Mepolizumab vs. Benralizumab: RR = 1.10 (0.70–1.80)<br>Mepolizumab vs. Dupilumab: RR = 1.20 (0.61–2.20)
Nopsopon et al. (2023)	***Eosinophils ≥ 300 cells/µL:***<br>Tezepelumab vs. Mepolizumab: RR = 0.83 (0.60–1.18)<br>Tezepelumab vs. Benralizumab: RR = 0.63 (0.46–0.85)*<br>Tezepelumab vs. Dupilumab: RR = 0.95 (0.62–1.45)<br><br>**SUCRA ranking:** Tezepelumab: 89%, Mepolizumab: 6%, Benralizumab: <1%, Dupilumab: 38%
Ando et al. (2022)	***Eosinophils ≥ 300 cells/µL***<br>Tezepelumab vs. Mepolizumab: RR = 0.960 (0.569–1.624)<br>Tezepelumab vs. Benralizumab: RR = 0.508 (0.356–0.725)*<br>Tezepelumab vs. Dupilumab: RR = 0.909 (0.581–1.429)<br>Dupilumab vs. Mepolizumab: RR = 1.056 (0.611–1.823)<br>Dupilumab vs. Benralizumab: RR = 0.558 (0.379–0.824)*<br>Benralizumab vs. Mepolizumab: RR = 1.891 (1.179–3.027)*<br><br>**SUCRA ranking:** Tezepelumab: 80.5%, Mepolizumab: 75.4%, Benralizumab: 15.2%, Dupilumab: 69.0%<br><br>***Eosinophils < 300 cells/µL***<br>Tezepelumab vs. Mepolizumab: RR = 0.939 (0.557–1.580)<br>Tezepelumab vs. Benralizumab: RR = 0.828 (0.586–1.169)<br>Tezepelumab vs. Dupilumab: RR = 0.714 (0.479–1.067)<br>Dupilumab vs. Mepolizumab: RR = 1.316 (0.751–2.300)<br>Dupilumab vs. Benralizumab: RR = 1.160 (0.778–1.731)<br>Benralizumab vs. Mepolizumab: RR = 1.134 (0.673–1.908)

	**SUCRA ranking:** Tezepelumab: 85%, Mepolizumab: 72.4%, Benralizumab: 55.6 %, Dupilumab: 33.3%<br><br>***Eosinophils ≥ 150 cells/µL***<br>Tezepelumab vs. Mepolizumab: RR = 1.004 (0.660–1.528)<br>Tezepelumab vs. Benralizumab: RR = 0.659 (0.512–0.850)*<br>Tezepelumab vs. Dupilumab: RR = 0.987 (0.699–1.398)<br>Dupilumab vs. Mepolizumab: RR = 1.017 (0.646–1.599)<br>Dupilumab vs. Benralizumab: RR = 0.668 (0.492–0.907)*<br>Benralizumab vs. Mepolizumab: RR = 1.522 (1.033–2.238)*<br><br>**SUCRA ranking:** Tezepelumab: 75.6%, Mepolizumab: 75.5%, Dupilumab: 73.4%, Benralizumab: 25.6 %<br><br>***Eosinophils < 150 cells/µL***<br>Tezepelumab vs. Mepolizumab: RR = 1.015 (0.487–2.115)<br>Tezepelumab vs. Benralizumab: RR = 0.847 (0.497–1.440)<br>Tezepelumab vs. Dupilumab: RR = 0.531 (0.302–0.939)*<br>Dupilumab vs. Mepolizumab: RR = 1.913 (0.887–4.114)<br>Dupilumab vs. Benralizumab: RR = 1.595 (0.898–2.847)<br>Benralizumab vs. Mepolizumab: RR = 1.198 (0.571–2.510)<br><br>**SUCRA ranking:** Tezepelumab: 79.9%, Mepolizumab: 77.4%, Benralizumab: 62.1 %, Dupilumab: 9.6 %
Bateman et al. (2022)	***Eosinophils ≥ 300 cells/µL:***<br>Dupilumab 200 mg vs. Omalizumab (150–375 mg): RR = 0.86 (0.49–1.51)<br>Dupilumab 300 mg vs. Omalizumab (150–375 mg): RR = 0.61 (0.35–1.09)<br>Dupilumab 200/300 mg vs. Omalizumab (150–375 mg): RR = 0.74 (0.50–1.10)
Menzies-Gow et al. (2022)	***Eosinophils ≥ 300 cells/µL***<br>Tezepelumab vs. Dupilumab:<br>200 mg: RR = 0.95 (0.47–1.92)<br>300 mg: RR = 1.08 (0.59–2.27)<br><br>Tezepelumab vs. Benralizumab: RR = 0.60 (0.34–1.13)<br>Tezepelumab vs. Mepolizumab: RR = 0.89 (0.42–1.95)<br>Tezepelumab vs. Reslizumab: RR = 0.70 (0.37–1.37)<br>Tezepelumab vs. Omalizumab: RR = 0.60 (0.29–1.30)<br><br>Dupilumab vs. Tezepelumab:<br>200 mg: RR = 1,05 (0,52–2,13)<br>300 mg: RR = 0,93 (0,44–1,86)<br><br>Dupilumab 200 mg vs. Dupilumab 300 mg: RR = 1,12 (0,59–2,27)<br>Dupilumab 300 mg vs Dupilumab 200 mg: RR = 0,89 (0,44–1,70)<br>Dupilumab vs. Benralizumab:<br>200 mg: RR = 0.64 (0.35–1.19)<br>300 mg: RR = 0.57 (0.30–1.04)<br><br>Dupilumab vs. Mepolizumab:<br>200 mg: RR = 0.94 (0.43–2.09)<br>300 mg: RR = 0.84 (0.37–1.77)<br><br>Dupilumab vs. Reslizumab:<br>200 mg: RR = 0.74 (0.38–1.46)<br>300 mg: RR = 0.66 (0.32–1.27)<br><br>Dupilumab vs. Omalizumab:<br>200 mg: RR = 0.64 (0.30–1.37)<br>300 mg: RR = 0.56 (0.26–1.19)<br><br>Benralizumab vs. Tezepelumab: RR = 1,67 (0,88–2,94)<br>Benralizumab vs. Dupilumab 200 mg: RR = 1,56 (0,84–2,86)

	Benralizumab vs. Dupilumab 300 mg: RR = 1,75 (0,96–3,33)<br>Benralizumab vs. Mepolizumab: RR = 1.47 (0.73–2.94)<br>Benralizumab vs. Reslizumab: RR = 1.16 (0.66–2.04)<br>Benralizumab vs. Omalizumab: RR = 0.99 (0.52–1.92)<br>Mepolizumab vs. Tezepelumab: RR = 1,12 (0,51–2,38)<br>Mepolizumab vs. Dupilumab 200 mg: RR = 1,06 (0,48–2,33)<br>Mepolizumab vs. Dupilumab 300 mg: RR = 1,19 (0,56–2,70)<br>Mepolizumab vs. Benralizumab: RR = 0,68 (0,34–1,37)<br>Mepolizumab vs. Reslizumab: RR = 0.79 (0.38–1.63)<br>Mepolizumab vs. Omalizumab: RR = 0.68 (0.31–1.55)<br>Reslizumab vs. Tezepelumab: RR = 1,43 (0,73–2,70)<br>Reslizumab vs. Dupilumab 200 mg: RR = 1,35 (0,68–2,63)<br>Reslizumab vs. Dupilumab 300 mg: RR = 1,52 (0,79–3,13)<br>Reslizumab vs. Benralizumab RR = 0,86 (0,49–1,51)<br>Reslizumab vs. Mepolizumab: RR = 1,27 (0,61–2,63)<br>Reslizumab vs. Omalizumab: RR = 0.86 (0.43–1.75)<br>Omalizumab vs. Tezepelumab: RR = 1,67 (0,77–3,45)<br>Omalizumab vs. Dupilumab 200 mg: RR = 1,56 (0,73–3,33)<br>Omalizumab vs. Dupilumab 300 mg: RR = 1,79 (0,84–3,85)<br>Omalizumab vs. Benralizumab: RR = 1,01 (0,52–1,91)<br>Omalizumab vs. Mepolizumab: RR = 1,47 (0,65–3,23)<br>Omalizumab vs. Reslizumab: RR = 1,16 (0,57–2,33)<br><br>***Eosinophils < 300 cells/µL***<br>Tezepelumab vs. Dupilumab 200 mg: RR = 0,69 (0,12–3,32)<br>Tezepelumab vs. Dupilumab 300 mg: RR = 0,61 (0,10–2,83)<br>Tezepelumab vs. Benralizumab: RR = 0,59 (0,10–2,62)<br>Dupilumab 200 mg vs. Tezepelumab: RR = 1,45 (0,30–8,33)<br>Dupilumab 200 mg vs. Dupilumab 300 mg: RR = 0,89 (0,22–3,54)<br>Dupilumab 200 mg vs. Benralizumab: RR = 0,86 (0,15–4,25)<br>Dupilumab 300 mg vs. Tezepelumab: RR = 1,64 (0,35–10,00)<br>Dupilumab 300 mg vs. Dupilumab 200 mg: RR = 1,12 (0,28–4,55)<br>Dupilumab 300 mg vs. Benralizumab: RR = 0,97 (0,18–4,92)<br>Benralizumab vs. Tezepelumab: RR = 1,69 (0,38–10,00)<br>Benralizumab vs. Dupilumab 200 mg: RR = 1,16 (0,24–6,67)<br>Benralizumab vs. Dupilumab 300 mg: RR = 1,03 (0,20–5,56)<br><br>***Eosinophils ≥ 150 cells/µL***<br>Tezepelumab vs. Dupilumab:<br>200 mg: RR = 0.84 (0.54, 1.33)<br>300 mg: RR = 0.91 (0.58, 1.44)<br>Tezepelumab vs. Benralizumab: RR = 0.63 (0.49, 0.82)*<br>Tezepelumab vs. Mepolizumab: RR = 0.94 (0.68, 1.30)<br>Tezepelumab vs. Omalizumab: RR = 0.63 (0.43, 0.94)*<br>Dupilumab vs. Tezepelumab:<br>200 mg: RR = 1.19 (0.75, 1.85)<br>300 mg: RR = 1.10 (0.69, 1.72)<br>Dupilumab 200 mg vs. Dupilumab 300 mg: RR = 1.08 (0.62, 1.92)<br>Dupilumab 300 mg vs Dupilumab 200 mg: RR = 0.93 (0.52, 1.62)<br>Dupilumab vs. Benralizumab:<br>200 mg: RR = 0.75 (0.48, 1.16)<br>300 mg: RR = 0.69 (0.45, 1.08)<br>Dupilumab vs. Mepolizumab:<br>200 mg: RR = 1.11 (0.69, 1.79)<br>300 mg: RR = 1.03 (0.64, 1.64)<br>Dupilumab vs. Omalizumab:<br>200 mg: RR = 0.75 (0.44, 1.27)<br>300 mg: RR = 0.69 (0.41, 1.17)<br>Benralizumab vs. Tezepelumab: RR = 1.59 (1.22, 2.04)*<br>Benralizumab vs. Dupilumab 200 mg: RR = 1.33 (0.86, 2.08)

	Benralizumab vs. Dupilumab 300 mg: RR = 1.45 (0.93, 2.22)<br>Benralizumab vs. Mepolizumab: RR = 1.47 (1.09, 2.00)*<br>Benralizumab vs. Omalizumab: RR = 1.00 (0.68, 1.45)<br>Mepolizumab vs. Tezepelumab: RR = 1.06 (0.77, 1.47)<br>Mepolizumab vs. Dupilumab 200 mg: RR = 0.90 (0.56, 1.45)<br>Mepolizumab vs. Dupilumab 300 mg: RR = 0.97 (0.61, 1.57)<br>Mepolizumab vs. Benralizumab: RR = 0.68 (0.50, 0.92)*<br>Mepolizumab vs. Omalizumab: RR = 0.67 (0.44, 1.04)<br>Omalizumab vs. Tezepelumab: RR = 1.59 (1.06, 2.33)*<br>Omalizumab vs. Dupilumab 200 mg: RR = 1.33 (0.79, 2.27)<br>Omalizumab vs. Dupilumab 300 mg: RR = 1.45 (0.85, 2.44)<br>Omalizumab vs. Benralizumab: RR = 1.00 (0.69, 1.47)<br>Omalizumab vs. Mepolizumab: RR = 1.49 (0.96, 2.27)<br><br>***Eosinophils < 150 cells/µL***<br>Tezepelumab vs. Dupilumab 200 mg: RR = 0.59 (0.33, 1.07)<br>Tezepelumab vs. Dupilumab 300 mg: RR = 0.48 (0.28, 0.84)*<br>Tezepelumab vs. Benralizumab: RR = 0.78 (0.47, 1.31)<br>Dupilumab 200 mg vs. Tezepelumab: RR = 1.69 (0.93, 3.03)<br>Dupilumab 200 mg vs. Dupilumab 300 mg: RR = 0.81 (0.43, 1.51)<br>Dupilumab 200 mg vs. Benralizumab: RR = 1.32 (0.74, 2.38)<br>Dupilumab 300 mg vs. Tezepelumab: RR = 2.08 (1.19, 3.57)*<br>Dupilumab 300 mg vs. Dupilumab 200 mg: RR = 1.23 (0.66, 2.33)<br>Dupilumab 300 mg vs. Benralizumab: RR = 1.64 (0.93, 2.86)<br>Benralizumab vs. Tezepelumab: RR = 1.28 (0.76, 2.13)<br>Benralizumab vs. Dupilumab 200 mg: RR = 0.76 (0.42, 1.36)<br>Benralizumab vs. Dupilumab 300 mg: RR = 0.61 (0.35, 1.08)
Ando et al. (2020)	***Eosinophils ≥ 300 cells/µL**:* Dupilumab vs. Benralizumab: RR = 0.58 (0.39–0.84)*<br>***Eosinophils 150–299 cells/µL:*** Dupilumab vs. Benralizumab: RR = 0.51 (0.29–0.92)*<br>***Eosinophils < 150 cells/µL:*** Dupilumab vs. Benralizumab: RR = 1.57 (0.73–2,82)
Edris et al. (2019)	***Eosinophils ≥ 300 cells/µL:***<br>Benralizumab vs. Dupilumab: RR = 0.439 (−1.120 to 2.070)<br>Benralizumab vs. Mepolizumab: RR = −0.019 (−1.300 to 1.380)<br>Benralizumab vs. Reslizumab: RR = 0.100 (−1.620 to 1.870)<br>Benralizumab vs. Tezepelumab: RR = 0.697 (−1.170 to 2.670)<br>Dupilumab vs. Benralizumab: RR = -0.439 (−2.070 to 1.120)<br>Dupilumab vs. Mepolizumab: RR = −0.472 (−2.330 to 1.530)<br>Dupilumab vs. Reslizumab: RR = −0.347 (−2.430 to 1.740)<br>Dupilumab vs. Tezepelumab: RR = 0.258 (−2.070 to 2.760)<br>Mepolizumab vs. Benralizumab: RR = 0.019 (-1.380 to 1.300)<br>Mepolizumab vs. Dupilumab: RR = 0.472 (−1.530 to 2.330)<br>Mepolizumab vs. Reslizumab: RR = 0.122 (−1.980 to 2.150)<br>Mepolizumab vs. Tezepelumab: RR = 0.709 (−1.580 to 2.980)<br>Reslizumab vs. Benralizumab: RR = −0,100 (− 1,870, 1,620)<br>Reslizumab vs. Dupilumab: RR = 0,347 (− 1,740, 2,430)<br>Reslizumab vs. Mepolizumab: RR = −0,122 (− 2,150, 1,980)<br>Reslizumab vs. Tezepelumab: RR = 0.604 (−1.810 to 3.090)
Busse et al. (2019)	***Eosinophils ≥ 400 cells/µL:***<br>Mepolizumab vs. Benralizumab: RR = 0.55 (0.35–0.87)*<br>Mepolizumab vs. Reslizumab: RR = 0.55 (0.36–0.85)*<br>Reslizumab vs. Benralizumab: RR = 1.00 (0.71–1.40)<br>***Eosinophils ≥ 300 cells/µL:***<br>Mepolizumab vs. Benralizumab: RR = 0.61 (0.37–0.99)*<br><br>***Eosinophils ≥ 150 cells/µL:***<br>Mepolizumab vs. Benralizumab: RR = 0.66 (0.49–0.89)*<br><br>***Unadjusted comparisons:***<br>Mepolizumab vs. Benralizumab: RR = 0.75 (0.56–1.00)<br>Mepolizumab vs. Reslizumab: RR = 0.89 (0.66–1.20)

	Benralizumab vs. Reslizumab: RR = 0.84 (0.63–1.13)
Cockle et al. (2017)	***Eosinophils ≥ 300 cells/µL:***<br>***Without population adjustment*:** Mepolizumab vs. Omalizumab: RR = 0.63 (0.45–0.89)<br>***With population adjustment:*** Mepolizumab vs. Omalizumab: RR = 0.66 (0.37–1.19)
Yan et al. (2019)	***GINA step 4/5 patients with ≥2 exacerbations*** (age ≥18 years)<br>Reslizumab vs. Mepolizumab: RR = 0.70 (0.53 to 0,95)*<br>***GINA step 4/5 patients with ≥2 exacerbations***<br>Reslizumab vs. Mepolizumab: RR = 0.77 (0.54 to 1,11)<br><br>***Overall***<br>Reslizumab vs. Mepolizumab: RR = 0.90 (0.38 to 2.04)<br><br>***Time to first exacerbation***<br>*(Overall)*<br>Reslizumab vs. Mepolizumab: RR = 1.22 (0.84 to 1,78)<br><br>***Any exacerbations***<br>*(Overall)*<br>Reslizumab vs. Mepolizumab: RR = 0.90 (0.38 to 2.04)

Source: Authors; RR: Rate Ratio; * significant.

3.1.3 Subgroup FeNO

Menzies-Gow et al. (2022)	***Overall***<br><br>***FeNO ≥25 ppb***<br>Tezepelumab vs. Dupilumab 200 mg: RR = 0.87 (0.50, 1.49)<br>Tezepelumab vs. Dupilumab 300 mg: RR = 0.80 (0.48, 1.33)<br>Dupilumab 200 mg vs. Tezepelumab: RR = 1.15 (0.67, 2.00)<br>Dupilumab 200 mg vs. Dupilumab 300 mg: RR = 0.92 (0.47, 1.81)<br>Dupilumab 300 mg vs. Tezepelumab: RR = 1.25 (0.75, 2.08)<br>Dupilumab 300 mg vs. Dupilumab 200 mg: RR = 1.09 (0.55, 2.13)<br><br>***Overall***<br><br>***FeNO ≥ 50 ppb***<br>Tezepelumab vs. Dupilumab 200 mg: RR = 0.87 (0.46, 1.63)<br>Tezepelumab vs. Dupilumab 300 mg: RR = 0.87 (0.49, 1.55)<br>Dupilumab 200 mg vs. Tezepelumab: RR = 1.15 (0.61, 2.17)<br>Dupilumab 200 mg vs. Dupilumab 300 mg: RR = 1.00 (0.49, 2.03)<br>Dupilumab 300 mg vs. Tezepelumab: RR = 1.15 (0.65, 2.04)<br>Dupilumab 300 mg vs. Dupilumab 200 mg: RR = 1.00 (0.49, 2.04)
Ando et al. (2022)	***FeNO ≥25 ppb***<br>Tezepelumab vs. Dupilumab: RR = 0.858 (0.567–1.306)<br>**SUCRA ranking:** Tezepelumab: 88.2%, Dupilumab: 61.9 %<br><br>***FeNO < 25 ppb***<br>Tezepelumab vs. Dupilumab: RR = 0.860 (0.554–1.341)<br>**SUCRA ranking:** Tezepelumab: 87.1%, Dupilumab: 58.7 %<br><br>***FeNO ≥ 50 ppb***<br>Tezepelumab vs. Dupilumab: RR = 0.870 (0.485–1.570)<br>**SUCRA ranking:** Tezepelumab: 83.9%, Dupilumab: 66.1 %<br><br>***FeNO < 50 ppb***<br>Tezepelumab vs. Dupilumab: RR = 0.843 (0.596–1.197)<br>**SUCRA ranking:** Tezepelumab: 91.6%, Dupilumab: 58.5 %

Source: Authors; RR: Rate Ratio; * significant.

3.1.4 Allergic asthma

Menzies-Gow et al. (2022)	***Overall***<br>Tezepelumab vs. Dupilumab:<br>200 mg: RR = 0.57 (0.18, 1.39)<br>300 mg: RR = 0.67 (0.20, 1.65)<br>Tezepelumab vs. Benralizumab: RR = 0.67 (0.20, 1.65)<br>Tezepelumab vs. Reslizumab: RR = 0.79 (0.29, 1.65)<br>Tezepelumab vs. Omalizumab: RR = 0.61 (0.24, 1.16)<br><br>Dupilumab vs. Tezepelumab:<br>200 mg: RR = 1.75 (0.72, 5.56)<br>300 mg: RR = 1.49 (0.61, 5.00)<br>Dupilumab 200 mg vs. Dupilumab 300 mg: RR = 1.15 (0.36, 3.85)<br>Dupilumab 300 mg vs Dupilumab 200 mg: RR = 0.87 (0.26, 2.77)<br><br>Dupilumab vs. Benralizumab:<br>200 mg: RR = 1.18 (0.36, 3.85)<br>300 mg: RR = 1.01 (0.31, 3.13)<br><br>Dupilumab vs. Reslizumab:<br>200 mg: RR = 1.37 (0.52, 3.85)<br>300 mg: RR = 1.18 (0.44, 3.33)<br><br>Dupilumab vs. Omalizumab:<br>200 mg: RR = 1.05 (0.42, 2.70)<br>300 mg: RR = 0.91 (0.36, 2.33)<br><br>Benralizumab vs. Tezepelumab: RR = 1.49 (0.61, 5.00)<br>Benralizumab vs. Dupilumab 200 mg: RR = 0.85 (0.26, 2.81)<br>Benralizumab vs. Dupilumab 300 mg: RR = 0.99 (0.32, 3.22)<br>Benralizumab vs. Reslizumab: RR = 1.18 (0.43, 3.33)<br>Benralizumab vs. Omalizumab: RR = 0.91 (0.35, 2.32)<br>Reslizumab vs. Tezepelumab: RR = 1.27 (0.61, 3.45)<br>Reslizumab vs. Dupilumab 200 mg: RR = 0.73 (0.26, 1.93)<br>Reslizumab vs. Dupilumab 300 mg: RR = 0.85 (0.30, 2.27)<br>Reslizumab vs. Benralizumab RR = 0.85 (0.30, 2.33)<br>Reslizumab vs. Omalizumab: RR = 0.77 (0.37, 1.58)<br>Omalizumab vs. Tezepelumab: RR = 1.64 (0.86, 4.17)<br>Omalizumab vs. Dupilumab 200 mg: RR = 0.95 (0.37, 2.38)<br>Omalizumab vs. Dupilumab 300 mg: RR = 1.10 (0.43, 2.78)<br>Omalizumab vs. Benralizumab: RR = 1.10 (0.43, 2.86)<br>Omalizumab vs. Reslizumab: RR = 1.30 (0.63, 2.70)

Source: Authors. RR: Rate Ratio; * significant.

3.1.5 CO-dependent Asthma

Phinyo et al. (2023)	Benralizumab vs. Dupilumab: Q8W: RR = 0.74 (0.36–1.51)<br>Benralizumab Q8W vs. Benralizumab Q4W: RR = 0.67 (0.37–1.20)<br><br>Benralizumab vs. Mepolizumab:<br>Q8W: RR = 0.44 (0.22–0.87)*<br>Q4W: RR = 0.66 (0.35–1.25)<br><br>Benralizumab vs. Tezepelumab:<br>Q8W: RR = 0.43 (0.21–0.90)*<br>Q4W: RR = 0.65 (0.33–1.30)<br><br>Benralizumab vs. Reslizumab:

	Q8W: RR = 0.37 (0.17–0.77)*<br>Q4W: RR = 0.55 (0.27–1.11)<br><br>Dupilumab vs. Benralizumab Q4W: RR = 0.90 (0.46–1.78)<br>Dupilumab vs. Mepolizumab: RR = 0.60 (0.34–1.06)<br>Dupilumab vs. Reslizumab: RR = 0.50 (0.26– 0.95)*<br>Mepolizumab vs. Tezepelumab: RR = 0.99 (0.55– 1,77)<br>Mepolizumab vs. Reslizumab: RR = 0.83 (0.45– 1,52)
Bourdin et al. (2020)	Benralizumab Q8W vs. Mepolizumab Q4W:<br>**Without population adjustment**: RR = 0.45 (0.24–0.82); p = 0.01<br>**With population adjustment**: RR = 0.56 (0.28–1.13); p = 0.1086<br><br>Benralizumab Q8W vs. Dupilumab Q2W:<br>**Without population adjustment**: RR = 0.75 (0.39–1.44); p = 0.38<br>**With population adjustment**: RR = 0.50 (0.20–1.28); p = 0.15

Source: Authors. Q4W: Every 4 weeks; Q8W: Every 8 weeks; RR: Rate Ratio; * significant.

3.2 Annual Rate of Exacerbations Requiring Hospital or Emergency Room Visits

3.2.1 Severe Uncontrolled Asthma (General Population)

Menzies-Gow et al. (2022)	Tezepelumab vs. Dupilumab 200/300 mg: RR = 0.36 (0.07–1.59)<br>Tezepelumab vs. Benralizumab: RR = 0.35 (0.08–1.16)<br>Tezepelumab vs. Mepolizumab: RR = 0.54 (0.13–2.00)<br>Tezepelumab vs. Reslizumab: RR = 0.29 (0.07–1.08)<br>Tezepelumab vs. Omalizumab: RR = 0.40 (0.10–1.55)<br>Dupilumab 200/300 mg vs. Tezepelumab: RR = 2,78 (0,63–14,29)<br>Dupilumab 200/300 mg vs. Benralizumab: RR = 1.00 (0.19–4.40)<br>Dupilumab 200/300 mg vs. Mepolizumab: RR = 1.52 (0.33–7.69)<br>Dupilumab 200/300 mg vs. Reslizumab: RR = 0.83 (0.18–4.12)<br>Dupilumab 200/300 mg vs. Omalizumab: RR = 1.15 (0.25–5.88)<br>Benralizumab vs. Tezepelumab: RR = 2,86 (0,86–12,50)<br>Benralizumab vs. Dupilumab 200/300 mg: RR = 1,00 (0,23–5,26)<br>Benralizumab vs. Mepolizumab: RR = 1.54 (0.45–6.67)<br>Benralizumab vs. Reslizumab: RR = 0.84 (0.24–3.56)<br>Benralizumab vs. Omalizumab: RR = 1.15 (0.35–5.00)<br>Mepolizumab vs. Tezepelumab: RR = 1,85 (0,50–7,69)<br>Mepolizumab vs. Dupilumab 200/300 mg: RR = 0,66 (0,13–3,04)<br>Mepolizumab vs. Benralizumab: RR = 0,65 (0,15–2,22)<br>Mepolizumab vs. Reslizumab: RR = 0.55 (0.14–2.05)<br>Mepolizumab vs. Omalizumab: RR = 0.75 (0.20–2.91)<br>Reslizumab vs. Tezepelumab: RR = 3,45 (0,93–14,29)<br>Reslizumab vs. Dupilumab 200/300 mg: RR = 1,20 (0,24–5,56)<br>Reslizumab vs. Benralizumab: RR = 1,19 (0,28–4,17)<br>Reslizumab vs. Mepolizumab: RR = 1,82 (0,49–7,14)<br>Reslizumab vs. Omalizumab: RR = 1.37 (0.37–5.56)<br>Omalizumab vs. Tezepelumab: RR = 2,50 (0,65–10,00)<br>Omalizumab vs. Dupilumab 200/300 mg: RR = 0,87 (0,17–3,96)<br>Omalizumab vs. Benralizumab: RR = 0,87 (0,20–2,89)<br>Omalizumab vs. Mepolizumab: RR = 1,33 (0,34–5,00)<br>Omalizumab vs. Reslizumab: RR = 0,73 (0,18–2,68)<br><br>**SUCRA ranking:** Tezepelumab: 95%, Mepolizumab: 71%, Omalizumab: 54%, Benralizumab: 45%, Dupilumab 200/300 mg: 45%, Reslizumab: 34%.

Bourdin et al. (2018)	Benralizumab Q8W vs. Mepolizumab (no matching adjustment): RR = 1.35 (0.78–2.36); p = 0.2837<br>Benralizumab Q8W vs. Mepolizumab (with matching adjustment): RR = 1.00 (0.57–1.75); p = 1.000

Source: Authors. Q4W: Every 4 weeks; Q8W: Every 8 weeks; RR: Rate Ratio; * significant.

3.2.2 Eosinophilic Asthma

Busse et al. (2019)	***Eosinophils ≥ 400 cells/µL:***<br>Mepolizumab vs. Reslizumab: RR = 1.24 (0.32–4.77)<br><br>***Eosinophils ≥ 300 cells/µL:***<br>Mepolizumab vs. Benralizumab: RR = 0.48 (0.11–2.08)<br><br>***Unadjusted comparisons:***<br>Mepolizumab vs. Benralizumab: RR = 0.54 (0.14–2.05)<br>Mepolizumab vs. Reslizumab: RR = 0.54 (0.24–1.24)<br>Reslizumab vs. Benralizumab: RR = 1.00 (0.27–3.69)
Cockle et al. (2017)	***Eosinophils ≥ 300 cells/µL:***<br>**Without population adjustment**: Mepolizumab vs. Omalizumab: RR = 0.58 (0.16–2.13)<br>**With population adjustment**: RR = 0.19 (0.02–2.32)
Yan et al. (2019)	***Eosinophils ≥ 300 cells/µL:***<br>***GINA step 4/5 patients with ≥2 exacerbations***<br>Reslizumab vs. Mepolizumab: RR = 1.22 (0.52 to 2,96)<br><br>***Overall***<br>Reslizumab vs. Mepolizumab: RR = 1.86 (0.89 to 4.07)

Source: Authors. RR: Rate Ratio; * significant.

3.3 Change in Pre-Bronchodilator FEV_1 (L)

3.3.1 Severe Uncontrolled Asthma (General Population)

Ando et al. (2022)	Tezepelumab vs. Mepolizumab: MD = 0.019 (−0.056 to 0.094)<br>Tezepelumab vs. Benralizumab: MD = 0.023 (−0.038 to 0.084)<br>Tezepelumab vs. Dupilumab: MD = 0.000 (−0.071 to 0.071)<br>Dupilumab vs. Mepolizumab: MD = 0.019 (−0.056 to 0.094)<br>Dupilumab vs. Benralizumab: MD = 0.023 (−0.038 to 0.084)<br>Benralizumab vs. Mepolizumab: MD = −0.004 (−0.070 to 0.062)<br><br>**SUCRA ranking:** Tezepelumab: 74.1%, Dupilumab: 74.0%, Mepolizumab: 54.2%, Benralizumab: 47.8%
Bateman et al. (2022)	***Week 12:***<br>Dupilumab 200 mg vs. Benralizumab Q8W: MD = 0.12 (−0.01 to 0.25)<br>Dupilumab 300 mg vs. Benralizumab Q8W: MD = 0.13 (0.00 to 0.26)<br>Dupilumab 200/300 mg vs. Benralizumab Q8W: MD = 0.12 (0.02 to 0.22)*<br>Dupilumab 200 mg vs. Mepolizumab: MD = 0.06 (−0.10 to 0.22)<br>Dupilumab 300 mg vs. Mepolizumab: MD = 0.10 (−0.05 to 0.26)<br>Dupilumab 200/300 mg vs. Mepolizumab: MD = 0.08 (−0.08 to 0.24)<br>Dupilumab 200 mg vs. Reslizumab Q4W: MD = 0.07 (−0.05 to 0.18)<br>Dupilumab 300 mg vs. Reslizumab Q4W: MD = 0.10 (−0.02 to 0.21)<br>Dupilumab 200/300 mg vs. Reslizumab Q4W: MD = 0.08 (−0.02 to 0.18)<br><br>***Week 24:***<br>Dupilumab 200 mg vs. Benralizumab Q8W: MD = 0.15 (0.03 to 0.27)

	Dupilumab 300 mg vs. Benralizumab Q8W: MD = 0.08 (−0.07 to 0.24)<br>Dupilumab 200/300 mg vs. Benralizumab Q8W: MD = 0.11 (0.01 to 0.21)*<br>Dupilumab 200 mg vs. Mepolizumab: MD = 0.09 (−0.03 to 0.21)<br>Dupilumab 300 mg vs. Mepolizumab: MD = 0.10 (−0.07 to 0.27)<br>Dupilumab 200/300 mg vs. Mepolizumab: MD = 0.09 (−0.05 to 0.24)<br>Dupilumab 200 mg vs. Reslizumab Q4W: MD = 0.15 (0.04 to 0.27)<br>Dupilumab 300 mg vs. Reslizumab Q4W: MD = 0.14 (0.02 to 0.25)<br>Dupilumab 200/300 mg vs. Reslizumab Q4W: MD = 0.14 (0.04 to 0.24)*
Ando et al. (2020)	Dupilumab vs. Benralizumab: MD = 0.032 (-0.047 to 0,111)
Bourdin et al. (2018)	***Baseline to Week 32:***<br>Benralizumab Q8W vs. Mepolizumab<br>*No adjustment:* MD = 0.04 (−0.05 to 0.13); p = 0.3572<br>*With adjustment:* MD = 0.03 (−0.06 to 0.12); p = 0.4898<br><br>***Baseline to the End of Study:***<br>Benralizumab Q8W vs. Mepolizumab<br>***No adjustment****:* MD = 0.02 (−0.06 to 0.10); p = 0.5768<br>***With adjustment****:* MD = 0.02 (−0.06 to 0.10); p = 0.6626
Henriksen et al. (2018)	Mepolizumab vs. Reslizumab: MD = −26.52 (−93.87 to 40.83)
Nachef et al. (2018)	Mepolizumab vs. Omalizumab: MD = 9.279 (−67.77 to 86.33); p = 1.000

Source: Authors. Q4W: Every 4 weeks; Q8W: Every 8 weeks; MD: Mean Difference; FEV: Forced Expiratory Volume; * significant.

3.3.2 Eosinophilic Asthma

Ando et al. (2022)	***Eosinophils ≥ 300 cells/μL***<br>Tezepelumab vs. Benralizumab: MD = 0.101 (0.010–0.191)*<br>Tezepelumab vs. Dupilumab: MD = −0.010 (−0.124 to 0.103)<br>Dupilumab vs. Benralizumab: MD = 0.111 (0.021–0.202)*<br><br>**SUCRA ranking:** Dupilumab: 85.5%, Tezepelumab: 80.5%, Benralizumab: 34.1%, Mepolizumab: Not determined<br><br>***Eosinophils < 300 cells/μL***<br>Tezepelumab vs. Benralizumab: MD = 0.022 (−0.079–0.122)<br>Tezepelumab vs. Dupilumab: MD = 0.022 (−0.073–0.117)<br>Dupilumab vs. Benralizumab: MD = −0.001 (−0.103–0.101)<br><br>**SUCRA ranking:** Tezepelumab: 77.5%, Benralizumab: 57.9%, Dupilumab: 57.5%, Mepolizumab: Not determined<br><br>***Eosinophils ≥ 150 cells/μL***<br>Tezepelumab vs. Benralizumab: MD = 0.053 (−0.018–0.124)<br>Tezepelumab vs. Dupilumab: MD = 0.023 (−0.064–0.110)<br>Dupilumab vs. Benralizumab: MD = 0.029 (−0.043–0.102)<br><br>**SUCRA ranking:** Dupilumab: 69.5%, Tezepelumab: 87.7%, Benralizumab: 42.9%, Mepolizumab: Not determined<br><br>***Eosinophils < 150 cells/μL***<br>Tezepelumab vs. Benralizumab: MD = −0.014 (−0.163–0.134)<br>Tezepelumab vs. Dupilumab: MD = −0.060 (−0.199–0.077)<br>Dupilumab vs. Benralizumab: MD = 0.046 (−0.098–0.191)<br><br>**SUCRA ranking:** Dupilumab: 83.6%, Benralizumab: 54.1%, Tezepelumab: 44.8%, Mepolizumab: Not determined
Bateman et al. (2022)	***Eosinophils ≥ 300 cells/μL***<br>***Week 24:***

	Dupilumab 200 mg vs. Omalizumab (150–375 mg): MD = 1.63 (−2.76 to 6.03)<br>Dupilumab 300 mg vs. Omalizumab (150–375 mg): MD = 4.32 (−0.14 to 8.79)<br>Dupilumab 200/300 mg vs. Omalizumab (150–375 mg): MD = 2.91 (−0.83 to 6.64)<br><br>***Week 52:***<br>Dupilumab 200 mg vs. Omalizumab (150–375 mg): MD = 5.51 (0.53 to 10.49)<br>Dupilumab 300 mg vs. Omalizumab (150–375 mg): MD = 8.41 (3.18 to 13.64)<br>Dupilumab 200/300 mg vs. Omalizumab (150–375 mg): MD = 6.83 (2.86 to 10.81)*
Ando et al. (2020)	***Eosinophils ≥ 300 cells/µL:***<br>Dupilumab vs. Benralizumab: MD = 0.106 (−0.007 to 0.218)
Busse et al. (2019)	***Eosinophils ≥ 400 cells/µL:***<br>Mepolizumab vs. Benralizumab: MD = −0.05 (−0.18 to 0.09)<br>Mepolizumab vs. Reslizumab: MD = 0.06 (−0.05 to 0.17)<br>Reslizumab vs. Benralizumab: MD = −0.11 (−0.20 to −0.01)*<br><br>***Eosinophils ≥ 300 cells/µL:***<br>Mepolizumab vs. Benralizumab: MD = 0.05 (−0.06 to 0.16)<br>Eosinophils ≥ 150 cells/µL:<br>Mepolizumab vs. Benralizumab: MD = 0.01 (−0.08 to 0.11)<br><br>***Unadjusted comparisons:***<br>Mepolizumab vs. Benralizumab: MD = −0.02 (−0.11 to 0.06)<br>Mepolizumab vs. Reslizumab: MD = −0.02 (−0.09 to 0.05)<br>Reslizumab vs. Benralizumab: MD = −0.00 (−0.08 to 0.07)
Iftikhar et al. (2018)	***Eosinophils ≥ 300 cells/µL***<br>Benralizumab vs. Dupilumab: MD = −0.03 (−0.12 to 0.05)<br>Benralizumab vs. Mepolizumab: MD = 0.03 (−0.04 to 0.10)<br>Benralizumab vs. Reslizumab: MD = −0.009 (−0.06 to 0.04)<br>Dupilumab vs. Benralizumab: MD = 0.03 (0.05 to 0,12)<br>Dupilumab vs. Mepolizumab: MD = 0.06 (−0.03 to 0.16)<br>Dupilumab vs. Reslizumab: MD = 0.02 (−0.06 to 0.11)<br>Mepolizumab vs. Benralizumab: MD = −0.03 (−0.10 to 0.04)<br>Mepolizumab vs. Dupilumab: MD = −0.06 (−0.16 to 0.03)<br>Mepolizumab vs. Reslizumab: MD = −0.03 (−0.10 to 0.02)<br>Reslizumab vs. Benralizumab = MD = 0.009 (−0.04 to 0.06)<br>Reslizumab vs. Dupilumab: MD = - 0.02 (- 0,11 to 0.06)<br>Reslizumab vs. Mepolizumab: MD = 0.03 (−0.02 to 0.10)
Cockle et al. (2017)	***Eosinophils ≥ 300 cells/µL***<br>***Without population adjustment**:* Mepolizumab vs. Omalizumab: MD = 0.24 (−3.61 to 4.10)<br>***With population adjustment**:* MD = −0.98 (−6.35 to 4.36)
Yan et al. (2019)	***Eosinophils ≥ 300 cells/µL***<br>***GINA step 4/5 patients with ≥2 exacerbations***<br>Reslizumab vs. Mepolizumab<br>4 weeks: MD = 0.14 (0.03 to 0.24)*<br>16 weeks: MD = 0.01 (−0.11 to 0.12)<br>24 weeks: MD = 0.04 (−0.08 to 0.15)<br><br>***Overall***<br>Reslizumab vs. Mepolizumab<br>4 weeks: MD = 0.00 (−0.07 to 0.06)<br>16 weeks: MD = 0.00 (−0.06 to 0.07)<br>24 weeks: MD = 0.00 (−0.09 to 0.09)<br><br>***Change in FEV$_1$ (% predicted)***<br>***Overall***<br>Reslizumab vs. Mepolizumab<br>4 weeks: RR = -1.63 (−4.29 to 1.02)<br>16 weeks: RR = -1.39 (−3.96 to 1.13)<br>24 weeks: RR = -2.04 (−5.47 to 1.37)

Source: Authors. MD: Mean Difference; FEV: Forced Expiratory Volume; * Significant.

3.4 Change in Pre-Bronchodilator FEV_1 (ml)

3.4.1 Eosinophilic Asthma

Akenroye et al. (2022)	***Eosinophils ≥ 300 cells/µL:***<br>Dupilumab vs. Benralizumab: MD = 76 (−9.9 to 160)<br>Mepolizumab vs. Benralizumab: MD = −8.5 (−100 to 83)<br>Mepolizumab vs. Dupilumab: MD = −85 (−190 to 19)<br><br>***Eosinophils 150–299 cells/µL:***<br>Dupilumab vs. Benralizumab: MD = −27 (−150 to 97)<br>Mepolizumab vs. Benralizumab: MD = −1.3 (−130 to 130)<br>Mepolizumab vs. Dupilumab: MD = 28 (−120 to 180)
Nopsopon et al. (2023)	***Eosinophils ≥ 300 cells/µL:***<br>Tezepelumab vs. Mepolizumab: MD = 66 (−33 to 170)<br>Tezepelumab vs. Benralizumab: MD = 62 (−22 to 150)<br>Tezepelumab vs. Dupilumab: MD = −20 (−120 to 78)<br><br>**SUCRA ranking:** Dupilumab: 95%, Tezepelumab: 34%, Mepolizumab: 2%, Benralizumab: <1%

Source: Authors. MD: Mean Difference; FEV: Forced Expiratory Volume; * Significant.

3.5 Change in Asthma Control Questionnaire (ACQ) Score

3.5.1 Uncontrolled Severe Asthma (General Population)

Ando et al. (2022)	Tezepelumab vs. Mepolizumab: MD = 0.080 (−0.097 to 0.258)<br>Tezepelumab vs. Benralizumab: MD = −0.069 (−0.214 to 0.075)<br>Tezepelumab vs. Dupilumab: MD = −0.160 (−0.337 to 0.018)<br>Dupilumab vs. Mepolizumab: MD = 0.240 (0.055 to 0.424)*<br>Dupilumab vs. Benralizumab: MD = 0.091 (−0.062 to 0.244)<br>Benralizumab vs. Mepolizumab: MD = 0.149 (−0.004 to 0.302)<br><br>**SUCRA ranking:** Mepolizumab: 94.5%, Tezepelumab: 74.3%, Benralizumab: 52.0%, Dupilumab: 28.9%
Nachef et al. (2018)	Mepolizumab vs. Omalizumab: MD = −0.02 (−0.53 to 0.49); p = 1.000
Henriksen et al. (2018)	Mepolizumab vs. Reslizumab: MD = −0.08 (−0.25 to 0.09)

Source: Authors. MD: Mean Difference; * Significant.

3.5.2 Eosinophilic Asthma

Akenroye et al. (2022)	***Eosinophils ≥ 300 cells/µL***<br>Dupilumab vs. Benralizumab: MD = −0.16 (−0.53 to 0.20)<br>Mepolizumab vs. Benralizumab: MD = −0.31 (−0.52 to −0.10)<br>Mepolizumab vs. Dupilumab: MD = −0.14 (−0.53 to 0.24)
Nopsopon et al. (2023)	***Eosinophils ≥ 300 cells/µL***<br>Tezepelumab vs. Mepolizumab: MD = 0.14 (−0.10 to 0.38)<br>Tezepelumab vs. Benralizumab: MD = −0.17 (−0.37 to 0.02)<br>Tezepelumab vs. Dupilumab: MD = −0.01 (−0.39 to 0.37)

	**SUCRA ranking:** Mepolizumab: 94%, Dupilumab: 21%, Tezepelumab: 9%, Benralizumab: <1%
Ando et al. (2022)	***Eosinophils ≥ 300 cells/µL***<br>Tezepelumab vs. Benralizumab: MD = −0.184 (−0.397 to 0.031)<br>**SUCRA ranking:** Tezepelumab: 97.7%, Benralizumab: 52.4%, Mepolizumab and Dupilumab: Not determined<br><br>***Eosinophils < 300 cells/µL***<br>Tezepelumab vs. Benralizumab: MD = −0.059 (−0.294–0.179)<br>**SUCRA ranking:** Tezepelumab: 84.0%, Benralizumab: 63.6%, Mepolizumab and Dupilumab: Not determined<br><br>***Eosinophils ≥ 150 cells/µL***<br>Tezepelumab vs. Benralizumab: MD = −0.184 (−0.352– −0.014)*<br>**SUCRA ranking:** Tezepelumab: 99.2%, Benralizumab: 50.9%, Mepolizumab and Dupilumab: Not determined
Busse et al. (2019)	***Eosinophils ≥ 400 cells/µL:***<br>Mepolizumab vs. Benralizumab: MD = −0.36 (−0.66 to −0.05)*<br>Mepolizumab vs. Reslizumab: MD = −0.39 (−0.66 to −0.12)*<br>Reslizumab vs Benralizumab: MD = 0.04 (−0.15 to 0.23)<br><br>***Eosinophils ≥ 300 cells/µL:***<br>Mepolizumab vs. Benralizumab: MD = −0.40 (−0.76 to −0.03)*<br><br>***Eosinophils ≥ 150 cells/µL:***<br>Mepolizumab vs. Benralizumab: MD = −0.33 (−0.54 to −0.11)*<br><br>***Unadjusted comparisons:***<br>Mepolizumab vs. Benralizumab: MD = −0.15 (−0.34 to 0.04)<br>Mepolizumab vs. Reslizumab: MD = −0.14 (−0.30 to 0.01)<br>Reslizumab vs. Benralizumab: MD = 0.00 (−0.16 to 0.15)
Iftikhar et al. (2018)	***Eosinophils ≥ 300 cells/µL***<br>Benralizumab vs. Dupilumab: MD = 0.02 (−0.18 to 0.24)<br>Benralizumab vs. Mepolizumab: MD = 0.13 (−0.02 to 0.29)<br>Benralizumab vs. Reslizumab: MD = −0.02 (−0.18 to 0.14)<br>Dupilumab vs. Benralizumab: MD = -0.02 (-0.24 to 0,18)<br>Dupilumab vs. Mepolizumab: MD = 0.11 (−0.11 to 0.34)<br>Dupilumab vs. Reslizumab: MD = -0.04 (−0.28 to 0.18)<br>Mepolizumab vs. Benralizumab: MD = −0.13 (−0.29 to 0.02)<br>Mepolizumab vs. Dupilumab: MD = −0.11 (−0.34 to 0.11)<br>Mepolizumab vs. Reslizumab: MD = −0.16 (−0.34 to 0.02)<br>Reslizumab vs. Benralizumab = MD = 0.02 (−0.14 to 0.18)<br>Reslizumab vs. Dupilumab: MD = 0.04 (- 0,18 to 0.28)<br>Reslizumab vs. Mepolizumab: MD = 0.16 (−0.02 to 0.34)

Source: Authors. MD: Mean Difference; * Significant.

3.6 Change in Asthma Quality of Life Questionnaire (AQLQ) Score

3.6.1 Uncontrolled Severe Asthma (General Population)

Ando et al. (2022)	Tezepelumab vs. Benralizumab: MD = 0.110 (−0.047 to 0.266)<br>Tezepelumab vs. Dupilumab: MD = 0.120 (−0.062 to 0.300)<br>Dupilumab vs. Benralizumab: MD = −0.010 (−0.177 to 0.159)<br><br>**SUCRA ranking:** Tezepelumab: 93.9%, Mepolizumab: not determined (ND), Benralizumab:

	54.3%, Dupilumab: 51.6%
Ando et al. (2020)	Dupilumab vs. Benralizumab: MD = 0.041 (−0.145 to 0.227)
Nachef et al. (2018)	Mepolizumab vs. Omalizumab: MD = −0.382 (−0.551 to −0.213); p < 0.001*
Henriksen et al. (2018)	Mepolizumab vs. Reslizumab: MD = 0.03 (−0.22 to 0.28)

Source: Authors; MD: Mean Difference; * Significant

3.6.2 Eosinophilic Asthma

Ando et al. (2022)	***Eosinophils ≥ 150 cells/µL***<br>Tezepelumab vs. Benralizumab: MD = 0.199 (0.018–0.382)*<br><br>**SUCRA ranking**: Tezepelumab: 99.2%, Mepolizumab: NE, Benralizumab: 50.8%, Dupilumab: NE
Ando et al. (2020)	***Eosinophils ≥ 300 cells/µL:***<br>Dupilumab vs. Benralizumab: MD = 0.042 (−0.220 to 0.304)
Iftikhar et al. (2018)	***Eosinophils ≥ 300 cells/µL:***<br>Benralizumab vs. Dupilumab: MD = -0.03 (−0.29 to 0.22)<br>Benralizumab vs. Mepolizumab: MD = -0.01 (−0.21 to 0.19)<br>Benralizumab vs. Reslizumab: MD = 0.002 (−0.18 to 0.19)<br>Dupilumab vs. Benralizumab: MD = 0.03 (-0.22 to 0,29)<br>Dupilumab vs. Mepolizumab: MD = 0.02 (−0.27 to 0.32)<br>Dupilumab vs. Reslizumab: MD = 0.03 (−0.24 to 0.31)<br>Mepolizumab vs. Benralizumab: MD = 0.01 (−0.19 to 0.21)<br>Mepolizumab vs. Dupilumab: MD = −0.02 (−0.32 to 0.27)<br>Mepolizumab vs. Reslizumab: MD = 0.01 (−0.22 to 0.24)<br>Reslizumab vs. Benralizumab = MD = 0.002 (−0.19 to 0.18)<br>Reslizumab vs. Dupilumab: MD = -0.03 (-0,31 to 0.24)<br>Reslizumab vs. Mepolizumab: MD = -0.01 (−0.24 to 0.22)

Source: Authors; MD: Mean Difference; * Significant.

3.7 Peak expiratory flow (PEF)

3.7.1 Severe Uncontrolled Asthma (General Population)

Nachef et al. (2018)	Mepolizumab vs. Omalizumab: MD = - 0,02 (- 0,531 to 0,491); p = 1.000

Source: Authors. MD: Mean Difference; PEF: Peak expiratory flow; * significant.

3.8 Reducing the CO

3.8.1 CO-dependent Asthma

Phinyo et al. (2023)	Benralizumab Q8W vs. Benralizumab Q4W:OR = 1.01 (0.56–1,81)<br><br>Benralizumab vs. Dupilumab:<br>Q8W: OR = 1.27 (0.56–2.87) / Q4W: OR = 1.26 (0.56–2.84)<br><br>Benralizumab vs. Mepolizumab:<br>Q8W: OR = 1.72 (0.70–4.22) / Q4W: OR = 1.71 (0.70–4.18)<br><br>Benralizumab vs. Tezepelumab:<br>Q8W: OR = 3.22 (1.35–7.68)* / Q4W: OR = 3.20 (1.34–7.60)*<br><br>Benralizumab vs. Reslizumab:<br>Q8W: OR = 3.35 (1.45–7.72)* / Q4W: OR = 3.33 (1.45–7.65)*<br><br>Dupilumab vs. Mepolizumab: OR = 1.36 (0.59–3.16)<br>Dupilumab vs. Tezepelumab: OR = 2.54 (1.12–5.73)*<br>Dupilumab vs. Reslizumab: OR = 2.64 (1.21–5.75)*<br>Mepolizumab vs. Tezepelumab: OR = 1.87 (0.76–4.56)<br>Mepolizumab vs. Reslizumab: OR = 1.94 (0.82–4.59)<br>Tezepelumab vs. Reslizumab: OR = 1.04 (0.45–2.39)
Bourdin et al. (2020)	**From baseline to week 24**<br>Benralizumab Q8W vs. Mepolizumab Q4W<br>*Without population adjustment:* MD = 20.10 (−7.83 to 48.03); p = 0.1584<br>*With population adjustment:* MD = 6.08 (−22.22 to 34.38); p = 0.6737<br><br>**From baseline to the end of the study**<br>Benralizumab Q8W vs. Mepolizumab Q4W<br>*Without population adjustment:* MD = −21.07 (−5.95 to 48.09); p = 0.1265<br>*With population adjustment:* MD = 5.06 (−22.39 to 32.52); p = 0.7177<br><br>**From baseline to week 24**<br>Benralizumab Q8W vs. Dupilumab Q2W<br>*Without population adjustment:* MD = 8.10 (−13.22 to 29.42); p = 0.46<br>*With population adjustment:* MD = −0.71 (−20.56 to 19.15); p = 0.94

Source: Authors; Q4W: Every 4 weeks; Q8W: Every 8 weeks ;OR: Odds Ratio; MD: Mean Difference; CO: Oral Corticosteroids; * significant.

3.9 Incidence of Adverse Events

3.9.1 Severe Uncontrolled Asthma – General Population

Ando et al. (2022)	Tezepelumab vs. Mepolizumab: OR = 1.009 (0.660–1.549)<br>Tezepelumab vs. Benralizumab: OR = 0.917 (0.647–1.297)<br>Tezepelumab vs. Dupilumab: OR = 0.964 (0.604–1.547)<br>Dupilumab vs. Mepolizumab: OR = 1.047 (0.655–1.674)<br>Dupilumab vs. Benralizumab: OR = 0.951 (0.637–1.416)<br>Benralizumab vs. Mepolizumab: OR = 1.101 (0.778–1.557)<br><br>**SUCRA ranking:** Tezepelumab: 66.5%, Mepolizumab: 68.4%, Benralizumab: 48.7%, Dupilumab: 57.7%
Ando et al. (2020)	Dupilumab vs. Benralizumab: OR = 1.023 (0.688–1.526)

Source: Authors; OR: Odds Ratio; * significant.

3.9.2 Eosinophilic Asthma

Cockle et al. (2017)	***Eosinophils ≥ 300 cells/µL:***<br>**Adverse events**<br>*Without population adjustment:* Mepolizumab vs. Omalizumab: OR = 0.79 (0,44 to 1,40)<br>*With population adjustment:* OR = 0.79 (0,31 to 1,91)<br><br>***Serious adverse events***<br>*Without population adjustment:* Mepolizumab vs. Omalizumab: OR = 0.68 (0,32 to 1,41)<br>*With population adjustment:* OR = 0.44 (0,11 to 1,57)<br><br>***Withdrawals due to adverse events***<br>*Without population adjustment:* Mepolizumab vs. Omalizumab: OR = 0.09 (0.003 to 0,84)<br>*With population adjustment:* OR = 0.05 (0,002 to 0,95)
Yan et al. (2019)	***Eosinophils ≥ 300 cells/µL:***<br>**Serious adverse events**: Reslizumab vs. Mepolizumab: RR = 1.65 (0.96–2.88)<br>**Discontinuations due to adverse events**: RR = 1.07 (0.35–3,50)<br>**Total adverse events**: Reslizumab vs. Mepolizumab: RR = 0.94 (0.85–1.04)
Akenroye et al. (2022)	***Eosinophils ≥ 300 cells/µL:***<br>Dupilumab vs. Benralizumab: OR = 1.40 (0.93–2.00)<br>Mepolizumab vs. Benralizumab: OR = 0.90 (0.60–1.30)<br>Mepolizumab vs. Dupilumab: OR = 0.65 (0.41–1.00)*

Source: Authors. MD: Mean Difference; Rate Ratio; OR: Odds Ratio; * significant.

3.10 Eosinophil Counts

3.10.1 Uncontrolled Severe Asthma (General Population)

Yan et al. (2019)	***Overall***<br>Reslizumab vs. Mepolizumab<br>4 weeks: MD = -165.23 (-255.97 to -73.92)*<br>16 weeks: MD = -117.03 (−197.01 to -37.11)*<br>24 weeks: MD = -202.17 (−312.23 to – 94.86)*

Source: Authors, MD: Mean Difference; * Significant.

4. METHODOLOGICAL QUALITY ASSESSMENT OF SYSTEMATIC REVIEWS: APPLICATION OF THE AMSTAR-2 TOOL

Domain	Phinyo et al. (2023)	Akenroye et al. (2022)	Nopsopon et al. (2023)	Ando et al. (2022)
**1 – Do the research questions and inclusion criteria for the review include the PICO components?**	**YES**	**YES**	**YES**	**YES**
	Rationale: The PICO was identified in the objective (considered the research question) of the study and complies with the inclusion criteria established by the reviewers. The PICO structure was not formally presented.	Rationale: The PICO was identified in the objective (considered the research question) of the study and complies with the inclusion criteria established by the reviewers. The PICO structure was not formally presented.	Rationale: The PICO was identified in the objective (considered the research question) of the study and complies with the inclusion criteria established by the reviewers. The PICO structure was not formally presented.	Rationale: The PICO was identified in the objective (considered the research question) of the study and complies with the inclusion criteria established by the reviewers. The PICO structure was formally presented.
**2 – Does the review report contain an explicit statement that the review methods were established before the review was carried out, and did the report justify any significant deviations from the protocol? (critical)**	**YES**	**YES**	**YES**	**NO**
	Rationale: The research protocol (CRD42023438828) was submitted, and no significant deviations from the protocol were reported.	Rationale: The research protocol (CRD42021232084) was presented, and no significant deviations from the protocol were reported.	Rationale: The research protocol (CRD42021232084) was presented, and no significant deviations from the protocol were reported.	Rationale: The registration of the research protocol was not presented, and there was no mention of any research protocol.
**3 – Have the authors of the review explained the selection of study designs for inclusion in the review?**	**YES**	**YES**	**YES**	**YES**
	Rationale: Randomized clinical trials (RCTs) were the study design of interest.	Rationale: Randomized clinical trials (RCTs) were the study design of interest.	Rationale: Phase IIb or III RCTs were the study designs of interest.	Rationale: Phase III or IIIb parallel-group RCTs were the study designs of interest.
**4 – Did the authors of the review use a comprehensive literature search strategy? (critical)**	**YES**	**YES**	**PARTIALLY YES**	**PARTIALLY YES**
	Rationale: Four databases were used; the search strategies were presented; a search was conducted in the gray literature, clinical trial	Rationale: Three databases were used; search strategies were presented in the supplementary material. A search was conducted	Rationale: Three databases were used, and the search strategies were presented in the supplementary material.	Rationale: Four databases were used, and search strategies were presented. A manual search was conducted, but sources for gray

	registries, and the reference lists of previously published systematic reviews and meta-analyses.	in gray literature, clinical trial registries, and reference lists of previously published systematic reviews and meta-analyses.	However, this was an update of a previous systematic review to include tezepelumab studies. No search in gray literature, clinical trial registries, or reference lists of prior studies was reported.	literature and clinical trial registries were not described. The references of the included studies were evaluated.
**5 – Did the authors of the review select the studies in duplicate?**	**YES**	**YES**	**YES**	**YES**
	Rationale: After duplicate removal, the remaining records were entered into ASReview software. The system was trained with relevant and irrelevant studies, and two authors reviewed 50% of the records according to prior consensus. They then independently assessed the included records for eligibility based on title, abstract, and full text, using Rayyan software.	Rationale: Two independent authors conducted the literature review using the Covidence systematic review software.	Rationale: Two authors updated the systematic review; however, it is not stated whether this was done independently.	Rationale: Two independent authors conducted the literature review.
**6 – Did the authors of the review extract data in duplicate?**	**YES**	**YES**	**YES**	**YES**
	Rationale: Two independent authors extracted the information of interest from the included studies.	Rationale: Two independent authors extracted the information of interest from the included studies.	Rationale: Two independent authors extracted the information of interest from the included studies.	Rationale: Two independent authors conducted the literature review.
**7 – Do the authors of the review provide a list of excluded studies and justify the exclusions? (critical)**	**YES**	**NO**	**NO**	**NO**
	Rationale: A list of excluded studies and the reasons for exclusion are reported in Supplementary Table E5.	Rationale: No list was provided presenting and justifying the studies excluded after full-text screening.	Rationale: No list was provided presenting and justifying the studies excluded after full-text screening.	Rationale: No list was provided presenting and justifying the studies excluded after full-text screening.
**8 – Do the review authors**	**YES**	**YES**	**YES**	**YES**

**describe the included studies in adequate detail?**	Rationale: The study described the population, intervention, comparators, outcomes, and type of study. In addition, it provided details of the population, intervention, and comparator (where necessary), reported the follow-up time of the included studies, and details of the outcomes of interest.	Rationale: The study described the population, intervention, comparators, outcomes, and type of study. In addition, it provided details of the population, intervention, and comparator (where necessary), reported the follow-up time of the included studies, and details of the outcomes of interest. These results are mainly presented in the supplementary material.	Rationale: The study described the population, intervention, comparators, outcomes, and type of study. It provided details of the population, the intervention, and the comparator (when necessary), reported the follow-up time of the included studies, and details of the outcomes of interest – most of which are available in the supplementary material.	Rationale: The study described the population, intervention, comparators, outcomes, and type of study. In addition, it provided details of the population, intervention, and comparator (where necessary), reported the follow-up time of the included studies, and details of the outcomes of interest. These results are presented mainly in the supplementary material.
**9 – Did the review authors use a satisfactory technique to assess the risk of bias (RoB) in individual studies that were included in the review? (critical)**	**YES**	**YES**	**YES**	**YES**
	Rationale: The ROB 2.0 tool was used. All the domains of interest were evaluated.	Rationale: The ROB 2.0 tool was used. All the domains of interest were evaluated.	Rationale: The tool used to assess the risk of bias was not explained. However, the results were presented in the supplementary material and described in the text of the review. All domains of interest were assessed.	Rationale: The ROB 2.0 tool was used. All the domains of interest were evaluated.
**10 – Did the authors of the review report the sources of funding for the studies included in the review?**	**NO**	**NO**	**NO**	**NO**
	Rationale: The sources of funding for the primary studies included in the review were not presented.	Rationale: The sources of funding for the primary studies included in the review were not presented.	Rationale: The funding sources of the primary studies included in the review were not presented.	Rationale: The funding sources of the primary studies included in the review were not presented.
**11 – If a meta-analysis was**	**YES**	**YES**	**YES**	**YES**

**carried out, did the authors of the review use appropriate methods for the statistical combination of results? (critical)**	Rationale: A frequentist network meta-analysis with random effects was carried out. For each treatment, the cumulative probability was calculated to derive the value of the area under the cumulative classification curve (SUCRA), which is finally used to classify the treatments.	Rationale: A Bayesian network meta-analysis (NMA) was carried out. Bayesian NMA models were fitted with generalized linear models. For each treatment, the cumulative probability was calculated to derive the value of the area under the cumulative rating curve (SUCRA), which is finally used to rank the treatments.	Rationale: A fixed-effect Bayesian meta-analysis was carried out to estimate rate ratios and mean differences in continuous outcomes. We calculated 95% credibility intervals with Monte Carlo algorithms. To compare and classify biologics, we used the SUCRA score and plotted the cumulative probability of leadership in different outcome improvements.	Rationale: A Bayesian network meta-analysis was performed following a robustly established methodology outlined by the National Institute for Health and Care Excellence (NICE), using the standard Bayesian model described by Dias et al., which considered inconsistency and heterogeneity between studies. Applying a non-informative prior distribution, the posterior distribution of the effect size was estimated using the Gibbs sampling technique based on the Monte Carlo method of Markov chains.
**12 – If a meta-analysis was carried out, did the review authors assess the potential impact of the risk of bias in individual studies on the results of the meta-analysis or other evidence synthesis?**	**YES**	**YES**	**YES**	**YES**
	Rationale: All included studies were classified as low risk of bias.	Rationale: Most of the included studies were classified as low risk of bias. In the sensitivity analyses, the risk of bias assessment was incorporated by excluding studies classified as having at least "some bias" in the	Rationale: The results of the sensitivity analyses, including the exclusion of studies with some risk of bias, were consistent with the primary analyses (Supplementary Table E4).	Rationale: Only one study was considered to present some concerns due to inadequate description of randomization. However, the authors did not carry out a sensitivity analysis to assess the impact of excluding

		specific outcome.		this study, as they did not consider it to pose a high risk of bias to the results.
**13 – Did the review authors consider the risk of bias in individual studies when interpreting/discussing the results of the review? (critical)**	**YES**	**YES**	**YES**	**YES**
	Rationale: All included studies were classified as low risk of bias.	Rationale: Most of the included studies were classified as low risk of bias. In the sensitivity analyses, the risk of bias assessment was incorporated by excluding studies classified as having at least "some bias" in the specific outcome.	Rationale: The results of the sensitivity analyses, including the exclusion of studies with some risk of bias, were consistent with the primary analyses (Supplementary Table E4).	Rationale: In cases of statistically significant heterogeneity between studies, relevant studies would be excluded. Only one study was considered to present some concerns due to inadequate description of randomization. However, the authors did not carry out a sensitivity analysis to assess the impact of excluding this study, as they did not consider it to pose a high risk of bias to the results.
**14 – Have the authors of the review provided a satisfactory explanation and discussion of any heterogeneity observed in the results of the review?**	**YES**	**YES**	**YES**	**YES**

	Rationale: Heterogeneity was assessed using Cochran's Q test and Higgins' I^2 statistic. In the discussion, it is noted that clinical and methodological heterogeneity limited the feasibility of additional analyses. However, heterogeneity is not described in the results.	Rationale: The Grading of Recommendations Assessment, Development and Evaluation (GRADE) approach was used to grade certainty in the NMA evidence, which was incorporated into the interpretation of results. Heterogeneity was considered in the GRADE domains. In the discussion, it is pointed out that by focusing on subgroups based on eosinophil counts, clinical heterogeneity was limited.	Rationale: The authors did not directly describe heterogeneity results. However, they discussed that the efficacy rating for antibodies across all endpoints in sensitivity analyses that included pooled intervention data was consistent with the primary analyses.	Rationale: Two studies (MENSA and MUSCA) compared mepolizumab with placebo, while SIROCCO, CALIMA, and ANDHI compared benralizumab with placebo. There was no statistically significant heterogeneity between the studies for the primary endpoint (AER), as shown by I^2 values of 0.0% ($p = 0.602$) for mepolizumab and 25.7% ($p = 0.260$) for benralizumab. No analyses of heterogeneity were identified for tezepelumab.
**15 – If they carried out quantitative synthesis, did the authors of the review carry out an adequate investigation of publication bias (small study bias) and discuss its likely impact on the results of the review? (critical)**	**NO**	**YES**	**YES**	**NO**
	Rationale: There was no analysis of publication bias, nor was it explained why this analysis was not carried out.	Rationale: An analysis of publication bias was carried out, and no significant evidence of publication bias was found.	Rationale: An analysis of publication bias was carried out, and no significant evidence of publication bias was found.	Rationale: There was no analysis of publication bias, nor was it explained why this analysis was not carried out.
**16 – Have the authors of the review reported any potential sources of conflict of interest, including any funding received to carry out the review?**	**YES**	**YES**	**YES**	**YES**
	Rationale: The authors reported potential conflicts of interest.	Rationale: The authors reported potential conflicts of interest and sources of funding.	Rationale: The authors reported potential conflicts of interest.	Rationale: The authors reported potential conflicts of interest.
**General methodological quality**	**LOW**	**LOW**	**LOW**	**CRITICALLY LOW**

Domain	Bateman et al. (2022)	Menzies-Gow et al. (2022)	Bourdin et al. (2020)	Ando et al. (2020)
**1 – Do the research questions and inclusion criteria for the review include the PICO components?**	**YES**	**YES**	**YES**	**YES**
	Rationale: The PICO was identified in the objective (considered the research question) of the study and complies with the inclusion criteria established by the reviewers. The PICO structure was formally presented.	Rationale: The PICO was identified in the objective (considered the research question) of the study and complies with the inclusion criteria established by the reviewers. The PICO structure was formally presented.	Rationale: The PICO was identified in the objective (considered the research question) of the study and complies with the inclusion criteria established by the reviewers. The PICO structure was not formally presented.	Rationale: The PICO was identified in the objective (considered the research question) of the study and complies with the inclusion criteria established by the reviewers. The PICO structure was not formally presented.
**2 – Does the review report contain an explicit statement that the review methods were established before the review was carried out, and did the report justify any significant deviations from the protocol? (critical)**	**NO**	**NO**	**NO**	**YES**
	Rationale: The registration of the research protocol was not presented, and there was no mention of the research protocol.	Rationale: The registration of the research protocol was not presented, and there was no mention of the research protocol.	Rationale: The registration of the research protocol was not presented, and there was no mention of the research protocol.	Rationale: The research protocol (UMIN-CTR no. UMIN000036256) was submitted, and there was no mention of significant deviations from this protocol.
**3 – Have the authors of the review explained the selection of study designs for inclusion in the review?**	**YES**	**YES**	**YES**	**YES**
	Rationale: Phase III or IIIb parallel-group RCTs were the study designs of interest.	Rationale: RCTs, systematic reviews, meta-analyses, and network meta-analyses were the study designs of interest.	Rationale: Randomized clinical trials (RCTs) were the study designs of interest.	Rationale: Randomized clinical trials (RCTs) were the study designs of interest.
**4 – Did the authors of the review use a comprehensive literature search strategy? (critical)**	**PARTIALLY YES**	**PARTIALLY YES**	**NO**	**YES**
	Rationale: Three databases were used, but the search strategies	Rationale: Three databases were used, the search strategies were	Rationale: The text does not describe whether a search was	Rationale: Four databases were used, the search strategies were

	were not presented. A manual search was carried out, but sources for gray literature and clinical trial registries were not described. The references of the included studies were evaluated.	presented, and the gray literature was searched. However, no searches were reported in clinical trial registries or in references of previously published studies.	carried out in several databases.	presented in the supplementary material, and a search was carried out in the gray literature, clinical trial registries, and references of previously published systematic reviews and meta-analyses.
**5 – Did the authors of the review select the studies in duplicate?**	**YES**	**YES**	**YES**	**YES**
	Rationale: Two independent authors conducted the literature review. It is not explicit in the text whether the full-text selection was conducted independently.	Rationale: Two independent authors carried out the abstract selection. It is not explicit in the text whether the full-text selection was conducted independently.	Rationale: Two independent authors carried out the abstract selection. It is not explicit in the text whether the full-text selection was conducted independently.	Rationale: Two independent authors conducted the literature review. It is not explicit in the text whether the full-text selection was conducted independently.
**6 – Did the authors of the review extract data in duplicate?**	**NO**	**NO**	**NO**	**NO**
	Rationale: It is not described whether data were extracted in duplicate by independent reviewers.	Rationale: It is not described whether data were extracted in duplicate by independent reviewers.	Rationale: It is not described whether data were extracted in duplicate by independent reviewers.	Rationale: It is not described whether data were extracted in duplicate by independent reviewers.
**7 – Do the authors of the review provide a list of excluded studies and justify the exclusions? (critical)**	**NO**	**NO**	**NO**	**NO**
	Rationale: There is no list available that presents and justifies the studies excluded after full reading.	Rationale: There is no list available that presents and justifies the studies excluded after full reading.	Rationale: There is no list available that presents and justifies the studies excluded after full reading.	Rationale: There is no list available that presents and justifies the studies excluded after full reading.
**8 – Do the review authors describe the included studies in adequate detail?**	**YES**	**YES**	**YES**	**YES**

	Rationale: The study described the population, intervention, comparators, outcomes, and type of study. It also provided details of the population, intervention, and comparator (where necessary), reported the follow-up time of the included studies, and details of the outcomes of interest. These results are presented mainly in the supplementary material.	Rationale: The study described the population, intervention, comparators, outcomes, and type of study. It also provided details of the population, intervention, and comparator (where necessary), reported the follow-up time of the included studies, and details of the outcomes of interest. These results are presented mainly in the supplementary material.	Rationale: The study described the population, intervention, comparators, outcomes, and type of study. It also provided details of the population, intervention, and comparator (where necessary), reported the follow-up time of the included studies, and details of the outcomes of interest. These results are presented mainly in the supplementary material.	Rationale: The study described the population, intervention, comparators, outcomes, and type of study. It also provided details of the population, intervention, and comparator (where necessary), reported the follow-up time of the included studies, and details of the outcomes of interest. These results are presented mainly in the supplementary material.
**9 – Did the review authors use a satisfactory technique to assess the risk of bias (RoB) in individual studies that were included in the review? (critical)**	**YES**	**YES**	**NO**	**YES**
	Rationale: The National Institute for Health and Care Excellence (NICE) quality appraisal checklist for quantitative intervention studies was used. All domains of interest were assessed.	Rationale: The National Institute for Health and Care Excellence (NICE) quality appraisal checklist for quantitative intervention studies was used. All domains of interest were assessed.	Rationale: The text did not discuss issues related to the methodological quality of the studies.	Rationale: The ROB 2.0 tool was used. All the domains of interest were evaluated.
**10 – Did the authors of the review report the sources of funding for the studies included in the review?**	**NO**	**NO**	**NO**	**NO**
	Rationale: The sources of funding for the primary studies included in the review were not presented.	Rationale: The sources of funding for the primary studies included in the review were not presented.	Rationale: The sources of funding for the primary studies included in the review were not presented.	Rationale: The sources of funding for the primary studies included in the review were not presented.
**11 – If a meta-analysis was carried out, did the authors of the review use appropriate methods for the statistical combination of results? (critical)**	**YES**	**YES**	**YES**	**YES**

	Rationale: Using frequentist meta-analysis, a pooled treatment effect was estimated for each biologic by pooling treatment effect estimates from all studies of the same biologic. Treatment effects were then compared using pairwise comparisons with a common comparator.	Rationale: Two types of indirect treatment comparisons were carried out – network meta-analyses (NMAs) and simulated treatment comparisons (STC) – to assess the robustness of treatment comparisons using fundamentally different approaches.	Rationale: A matched adjusted indirect comparison (MAIC) was performed to assess the relative effects of three biologic treatments on OCS reduction.	Rationale: An indirect treatment comparison was performed using a Bayesian approach to estimate differences in efficacy and safety between dupilumab and benralizumab.
**12 – If a meta-analysis was carried out, did the review authors assess the potential impact of the risk of bias in individual studies on the results of the meta-analysis or other evidence synthesis?**	**NO**	**NO**	**NO**	**YES**
	Rationale: The methodological quality of the included studies was assessed, but the potential impact of bias was not discussed.	Rationale: The methodological quality of the included studies was assessed, but the potential impact of bias was not discussed. The results are only available in the supplementary material.	Rationale: The text did not discuss issues related to the methodological quality of the studies.	Rationale: Assessment using the Cochrane risk of bias tool revealed a low risk of bias for all included studies.
**13 – Did the review authors consider the risk of bias in individual studies when interpreting/discussing the results of the review? (critical)**	**NO**	**NO**	**NO**	**YES**
	Rationale: The methodological quality of the included studies was assessed, but the potential impact of bias was not discussed.	Rationale: The methodological quality of the included studies was assessed, but the potential impact of bias was not discussed. The results are only available in the supplementary material.	Rationale: The text did not discuss issues related to the methodological quality of the studies.	Rationale: The Cochrane risk of bias assessment indicated low risk for all included studies, and this was considered in the interpretation.
**14 – Have the authors of the review provided a satisfactory explanation and discussion of any heterogeneity observed in the results of the review?**	**NO**	**YES**	**NO**	**YES**
	Rationale: The text did not discuss issues related to heterogeneity of the included studies.	Rationale: Heterogeneity was observed in eligibility criteria and patient characteristics. Its impact was considered low due to consistency in the analyses.	Rationale: The text did not discuss issues related to heterogeneity of the included studies.	Rationale: The authors did not directly describe heterogeneity results but stated that predefined inclusion/exclusion criteria were adapted to address clinical or methodological heterogeneity and ensure the quality of the indirect comparison.
**15 – If they carried out**	**NO**	**NO**	**NO**	**YES**

**quantitative synthesis, did the authors of the review carry out an adequate investigation of publication bias (small study bias) and discuss its likely impact on the results of the review? (critical)**	Rationale: The text did not discuss issues related to possible publication bias.	Rationale: There was no analysis of publication bias, nor was it explained why this analysis was not carried out.	Rationale: There was no analysis of publication bias, nor was it explained why this analysis was not carried out.	Rationale: To minimize publication bias, a review of reference lists and a manual search for relevant articles were conducted.
**16 – Have the authors of the review reported any potential sources of conflict of interest, including any funding received to carry out the review?**	**YES**	**YES**	**YES**	**YES**
	Rationale: The authors reported potential conflicts of interest and sources of funding.	Rationale: The authors reported potential conflicts of interest.	Rationale: The authors reported potential conflicts of interest and sources of funding.	Rationale: The authors reported potential conflicts of interest and sources of funding.
**General methodological quality**	**CRITICALLY LOW**	**CRITICALLY LOW**	**CRITICALLY LOW**	**LOW**

Domain	Ramonell and Iftikhar (2020)	Edris et al. (2019)	Busse et al. (2019)	Yan et al. (2019)
**1 – Do the research questions and inclusion criteria for the review include the PICO components?**	**YES**	**YES**	**YES**	**YES**
	Rationale: The PICO was identified in the objective (considered the research question) of the study and follows the inclusion criteria established by the reviewers. The PICO structure was not formally presented.	Rationale: The PICO was identified in the objective (considered the research question) of the study and follows the inclusion criteria established by the reviewers. The PICO structure was not formally presented.	Rationale: The PICO was identified in the objective (considered the research question) of the study and follows the inclusion criteria established by the reviewers. The PICO structure was not formally presented.	Rationale: The PICO was identified in the objective (considered the research question) of the study and follows the inclusion criteria established by the reviewers. The PICO structure was not formally presented.
**2 – Does the review report contain an explicit statement that the review methods were established before the review was carried out, and did the report justify any significant deviations from the protocol? (critical)**	**NO**	**YES**	**NO**	**PARTIALLY YES**
	Rationale: The registration of the research protocol was not presented, and there was no mention of the research protocol.	Rationale: The research protocol (CRD42019127706) was presented, and there was no mention of significant deviations from this protocol.	Rationale: The registration of the research protocol was not presented, and there was no mention of the research protocol.	Rationale: The research protocol was not presented, but a protocol developed by Precision Xtract was mentioned.
**3 – Have the authors of the review explained the selection of study designs for inclusion in the review?**	**YES**	**YES**	**YES**	**YES**
	Rationale: Randomized clinical trials (RCTs) were the study designs of interest.	Rationale: Phase II and III RCTs were the study designs of interest.	Rationale: Randomized clinical trials (RCTs) were the study designs of interest.	Rationale: Randomized clinical trials (RCTs) were the study designs of interest.
**4 – Did the authors of the review use a comprehensive literature search strategy? (critical)**	**YES**	**PARTIALLY YES**	**PARTIALLY YES**	**YES**
	Rationale: Two databases were used; the search strategies were presented; a search was carried out in the gray literature, in clinical trial registries, and in references of previously published systematic reviews and meta-analyses.	Rationale: Two databases were used; the search strategies were not presented. A manual search was carried out, but the search sources for gray literature and clinical trial registries were not described. The references of the included studies were evaluated.	Rationale: The main data source for this ITC was a recent Cochrane review on anti–IL-5 pathway therapies in severe asthma. Additional searches were conducted in January 2018 to identify any new publications or datasets.	Rationale: Three databases were used, and the search strategies were presented in the supplementary material.
**5 – Did the authors of the review select the studies in**	**YES**	**YES**	**NO**	**YES**
	Rationale: Two independent	Rationale: Two independent	Rationale: Data from eligible	Rationale: Two independent

**duplicate?**	authors conducted the literature review.	authors conducted the literature review.	studies were extracted by one study member and independently reviewed by another.	authors conducted the literature review.
**6 – Did the authors of the review extract data in duplicate?**	**YES**	**YES**	**NO**	**YES**
	Rationale: Two independent authors extracted the information of interest from the included studies.	Rationale: Two independent authors conducted the literature review.	Rationale: Data were extracted by one study member and independently reviewed by another.	Rationale: Two independent authors extracted the information of interest from the included studies.
**7 – Do the authors of the review provide a list of excluded studies and justify the exclusions? (critical)**	**YES**	**NO**	**NO**	**NO**
	Rationale: A list of excluded studies and the reasons for exclusion are reported in the supplementary material.	Rationale: There is no list available that presents and justifies the studies excluded after full reading.	Rationale: There is no list available that presents and justifies the studies excluded after full reading.	Rationale: There is no list available that presents and justifies the studies excluded after full reading.
**8 – Do the review authors describe the included studies in adequate detail?**	**YES**	**YES**	**YES**	**YES**
	Rationale: The study described the population, intervention, comparators, outcomes, and study type. It provided details of the population, intervention, and comparator (when applicable), reported the follow-up time of the included studies, and the outcomes of interest.	Rationale: The study described the population, intervention, comparators, outcomes, and study type. It provided additional details and reported follow-up and outcomes, mainly in the supplementary material.	Rationale: The study described the population, intervention, comparators, outcomes, and study type. It provided details of the population, intervention, comparator, follow-up time, and outcomes, mainly in the supplementary material.	Rationale: The study described the population, intervention, comparators, outcomes, and study type. It provided details of the population, intervention, comparator, follow-up time, and outcomes, mainly in the supplementary material.
**9 – Did the review authors use a satisfactory technique to assess the risk of bias (RoB) in individual studies that were included in the review? (critical)**	**YES**	**YES**	**NO**	**YES**
	Rationale: The studies were assessed for “study quality” based on modified Cochrane methods.	Rationale: The risk of bias was assessed using the Cochrane risk of bias tool for randomized clinical trials.	Rationale: The text did not discuss issues related to the methodological quality of the studies.	Rationale: The risk of bias was assessed using the Cochrane risk of bias tool for randomized clinical trials.
**10 – Did the authors of the**	**NO**	**NO**	**NO**	**NO**

**review report the sources of funding for the studies included in the review?**	Rationale: The sources of funding for the primary studies included in the review were not presented.	Rationale: The sources of funding for the primary studies included in the review were not presented.	Rationale: The sources of funding for the primary studies included in the review were not presented.	Rationale: The funding sources of the primary studies included in the review were not presented.
**11 – If a meta-analysis was carried out, did the authors of the review use appropriate methods for the statistical combination of results? (critical)**	**YES**	**YES**	**YES**	**YES**
	Rationale: A network meta-analysis was conducted comparing the efficacy of FDA-approved monoclonal antibody therapies in preventing exacerbations in patients with severe eosinophilic asthma.	Rationale: A network meta-analysis of RCTs comparing monoclonal antibodies in type 2 asthma was carried out.	Rationale: An indirect treatment comparison (ITC) was performed for licensed doses of mepolizumab, benralizumab, and reslizumab, according to baseline eosinophil counts.	Rationale: A systematic review and network meta-analysis (NMA) was conducted to evaluate the comparative efficacy and safety of these treatments in patients with inadequately controlled moderate to severe eosinophilic asthma.
**12 – If a meta-analysis was carried out, did the review authors assess the potential impact of the risk of bias in individual studies on the results of the meta-analysis or other evidence synthesis?**	**NO**	**YES**	**NO**	**YES**
	Rationale: The methodological quality of the included studies was assessed, but the potential impact of bias was not discussed.	Rationale: The methodological quality of the included studies was assessed, and they presented a limited risk of bias.	Rationale: The text did not discuss issues related to the methodological quality of the studies.	Rationale: The methodological quality of the included studies was assessed. The studies presented a low risk of bias.
**13 – Did the review authors consider the risk of bias in individual studies when interpreting/discussing the results of the review? (critical)**	**NO**	**YES**	**NO**	**YES**
	Rationale: The methodological quality of the included studies was assessed, but the potential impact of bias was not discussed.	Rationale: The methodological quality of the included studies was assessed. The studies presented a limited risk of bias.	Rationale: The text did not discuss issues related to the methodological quality of the studies.	Rationale: The methodological quality of the included studies was assessed. The studies presented a low risk of bias.
**14 – Have the authors of the review provided a satisfactory explanation and discussion of any heterogeneity observed in the results of the review?**	**YES**	**NO**	**YES**	**YES**
	Rationale: Heterogeneity was assessed using the I^2 index in direct pairwise analyses.	Rationale: The text did not discuss issues related to heterogeneity in the included studies.	Rationale: No heterogeneity was found between mepolizumab and reslizumab. High heterogeneity was observed among benralizumab studies, likely not due to study duration.	Rationale: Heterogeneity was addressed in the limitations section of the study.

**15 – If they carried out quantitative synthesis, did the authors of the review carry out an adequate investigation of publication bias (small study bias) and discuss its likely impact on the results of the review? (critical)**	**YES**	**NO**	**NO**	**YES**
	Rationale: An analysis of publication bias was conducted using a funnel plot adjusted by comparison.	Rationale: The text did not mention analysis of publication bias.	Rationale: There was no analysis of publication bias, nor was it explained why it was not conducted.	Rationale: An analysis of publication bias was carried out and no significant evidence of bias was found.
**16 – Have the authors of the review reported any potential sources of conflict of interest, including any funding received to carry out the review?**	**YES**	**YES**	**NO**	**YES**
	Rationale: The authors reported potential conflicts of interest and sources of funding.	Rationale: The authors reported potential conflicts of interest and sources of funding.	Rationale: The authors did not report potential conflicts of interest or funding sources.	Rationale: The authors reported potential conflicts of interest and sources of funding.
**General methodological quality**	**CRITICALLY LOW**	**CRITICALLY LOW**	**CRITICALLY LOW**	**LOW**

Domain	Iftikhar et al. (2018)	Nachef et al. (2018)	Bourdin et al. (2018)	Henriksen et al. (2018)
**1 – Do the research questions and inclusion criteria for the review include the PICO components?**	**YES**	**YES**	**YES**	**YES**
	Rationale: The PICO was identified in the objective (considered as the research question) of the study and complies with the inclusion criteria established by the reviewers. The PICO structure was not formally presented.	Rationale: The PICO was identified in the objective (considered as the research question) of the study and complies with the inclusion criteria established by the reviewers. The PICO structure was formally presented.	Rationale: The PICO was identified in the objective (considered as the research question) of the study and complies with the inclusion criteria established by the reviewers. The PICO structure was formally presented in the supplementary material.	Rationale: The PICO was identified in the objective (considered as the research question) of the study and complies with the inclusion criteria established by the reviewers. The PICO structure was formally presented.
**2 – Does the review report contain an explicit statement that the review methods were established before the review was carried out, and did the report justify any significant deviations from the protocol? (critical)**	**NO**	**NO**	**NO**	**NO**
	Rationale: The registration of the research protocol was not presented, and there was no mention of the research protocol.	Rationale: The registration of the research protocol was not presented, and there was no mention of the research protocol.	Rationale: The registration of the research protocol was not presented, and there was no mention of the research protocol.	Rationale: The registration of the research protocol was not presented, and there was no mention of the research protocol.
**3 – Have the authors of the review explained the selection of study designs for inclusion in the review?**	**YES**	**YES**	**YES**	**YES**
	Rationale: English-only randomized clinical trials (RCTs) were the study designs of interest.	Rationale: Randomized clinical trials (RCTs) were the study designs of interest.	Rationale: Randomized clinical trials (RCTs) were the study designs of interest.	Rationale: Systematic reviews and randomized clinical trials (RCTs) were the study designs of interest.
**4 – Did the authors of the review use a comprehensive literature search strategy? (critical)**	**PARTIALLY YES**	**YES**	**YES**	**YES**
	Rationale: One database was used; the search strategies were	Rationale: Five databases were used; the search strategies were	Rationale: Four databases were used; the search strategies were	Rationale: Three databases were used; the search strategies were

	presented in the supplementary material. A search was conducted in the gray literature, clinical trial registries, and references of previously published systematic reviews and meta-analyses.	not presented. A manual search was carried out, but sources for gray literature and clinical trial registries were not described. References of included studies were evaluated.	presented in the supplementary material. A search was conducted in the gray literature, clinical trial registries, and references of previously published systematic reviews and meta-analyses.	presented in the supplementary material. A search was conducted in the gray literature, clinical trial registries, and references of previously published systematic reviews and meta-analyses.
**5 – Did the authors of the review select the studies in duplicate?**	**NO**	**YES**	**YES**	**YES**
	Rationale: Data from eligible studies were extracted by one study member and independently reviewed by a different member.	Rationale: Two independent authors conducted the literature review.	Rationale: Two independent authors conducted the full literature review.	Rationale: Two independent authors conducted a comprehensive literature review.
**6 – Did the authors of the review extract data in duplicate?**	**NO**	**YES**	**YES**	**YES**
	Rationale: Data from eligible studies were extracted by one study member and independently reviewed by a different member.	Rationale: Two independent authors conducted the literature review.	Rationale: Two independent reviewers carried out screening and data extraction activities, with discrepancies reconciled by a third independent reviewer.	Rationale: Two independent authors extracted the information of interest from the included studies.
**7 – Do the authors of the review provide a list of excluded studies and justify the exclusions? (critical)**	**NO**	**NO**	**NO**	**NO**
	Rationale: There is no list available that presents and justifies the studies excluded after full reading.	Rationale: There is no list available that presents and justifies the studies excluded after full reading.	Rationale: There is no list available that presents and justifies the studies excluded after full reading.	Rationale: There is no list available that presents and justifies the studies excluded after full reading.
**8 – Do the review authors describe the included studies in adequate detail?**	**YES**	**YES**	**YES**	**YES**

	Rationale: The study described the population, intervention, comparators, outcomes, and study type. It also provided details of the population, intervention, and comparator (when necessary), reported the follow-up time of the included studies, and details of the outcomes of interest.	Rationale: The study described the population, intervention, comparators, outcomes, and study type. It also provided details of the population, intervention, and comparator (when necessary), reported the follow-up time of the included studies, and details of the outcomes of interest.	Rationale: The study described the population, intervention, comparators, outcomes, and study type. It also provided details of the population, intervention, and comparator (when necessary), reported the follow-up time of the included studies, and details of the outcomes of interest. These results are presented mainly in the supplementary material.	Rationale: The study described the population, intervention, comparators, outcomes, and study type. It also provided details of the population, intervention, and comparator (when necessary), reported the follow-up time of the included studies, and details of the outcomes of interest. These results are presented mainly in the supplementary material.
**9 – Did the review authors use a satisfactory technique to assess the risk of bias (RoB) in individual studies that were included in the review? (critical)**	**YES**	**YES**	**YES**	**YES**
	Rationale: The studies were assessed for "study quality" based on modified Cochrane methods.	Rationale: The quality and risk of bias of the selected studies were evaluated using the Jadad score (Oxford quality scoring system).	Rationale: The risk of bias was assessed using a NICE checklist. Sources of clinical heterogeneity were summarized and assessed. Each study was classified as having a high, low, or unclear risk of bias.	Rationale: The risk of bias was assessed using the Cochrane risk of bias tool for randomized controlled trials and systematic reviews.
**10 – Did the authors of the review report the sources of funding for the studies included in the review?**	**NO**	**NO**	**NO**	**NO**
	Rationale: The sources of funding for the primary studies included in the review were not presented.	Rationale: The sources of funding for the primary studies included in the review were not presented.	Rationale: The sources of funding for the primary studies included in the review were not presented.	Rationale: The sources of funding for the primary studies included in the review were not presented.
**11 – If a meta-analysis was carried out, did the authors of the review use appropriate methods for the statistical combination of results? (critical)**	**YES**	**YES**	**YES**	**YES**

	Rationale: A systematic review with network meta-analysis was carried out to synthesize data on the relative efficacy of benralizumab, dupilumab, mepolizumab, and reslizumab through frequentist analysis.	Rationale: A network meta-analysis was conducted to examine the comparative efficacy of omalizumab and mepolizumab in the treatment of severe asthma.	Rationale: A matched adjusted indirect comparison (MAIC) of benralizumab versus monoclonal antibodies targeting IL-5 was performed in patients with severe uncontrolled asthma and eosinophilic phenotype.	Rationale: A systematic review with meta-analysis was conducted to evaluate the efficacy, adverse events, and comparisons between mepolizumab and reslizumab for treating severe eosinophilic asthma.
**12 – If a meta-analysis was carried out, did the review authors assess the potential impact of the risk of bias in individual studies on the results of the meta-analysis or other evidence synthesis?**	**YES**	**YES**	**NO**	**YES**
	Rationale: The methodological quality of the included studies was assessed, and the potential impact of bias was discussed.	Rationale: The methodological quality of the included studies was assessed, and the potential impact of bias was discussed.	Rationale: The methodological quality of the included studies was assessed, but the potential impact of bias was not discussed.	Rationale: In general, the risk of bias assessed using the Cochrane Risk of Bias Tool was considered low. Two studies did not address random sequence generation or allocation concealment, and one did not address blinding of outcome assessment.
**13 – Did the review authors consider the risk of bias in individual studies when interpreting/discussing the results of the review? (critical)**	**YES**	**YES**	**NO**	**YES**
	Rationale: The methodological quality of the included studies was assessed, and the potential impact of bias was discussed.	Rationale: The methodological quality of the included studies was assessed, and the potential impact of bias was discussed.	Rationale: The methodological quality of the included studies was assessed, but the potential impact of bias was not discussed.	Rationale: In general, the risk of bias was considered low, despite some limitations related to randomization and blinding.
**14 – Have the authors of the review provided a satisfactory explanation and discussion of any heterogeneity observed in the results of the review?**	**NO**	**YES**	**YES**	**YES**
	Rationale: The text did not discuss issues related to heterogeneity of the included studies.	Rationale: Heterogeneity tests were conducted for the included studies.	Rationale: Sources of clinical heterogeneity were summarized and evaluated. To limit heterogeneity, only trials with	Rationale: The statistical test for heterogeneity (Cochran's Q) was performed, and heterogeneity was described using the I^2

			exacerbations as a primary endpoint were included.	statistic. Differences in design and study characteristics were examined.
**15 – If they carried out quantitative synthesis, did the authors of the review carry out an adequate investigation of publication bias (small study bias) and discuss its likely impact on the results of the review? (critical)**	**YES**	**NO**	**NO**	**NO**
	Rationale: An analysis of publication bias was carried out using a funnel plot adjusted by comparison.	Rationale: The text did not discuss issues related to possible publication bias.	Rationale: The text did not discuss issues related to possible publication bias.	Rationale: The text did not discuss issues related to possible publication bias.
**16 – Have the authors of the review reported any potential sources of conflict of interest, including any funding received to carry out the review?**	**YES**	**YES**	**YES**	**YES**
	Rationale: The authors reported potential conflicts of interest and sources of funding.	Rationale: The authors reported potential conflicts of interest and sources of funding.	Rationale: The authors reported potential conflicts of interest and sources of funding.	Rationale: The authors reported potential conflicts of interest and sources of funding.
**General methodological quality**	**CRITICALLY LOW**	**CRITICALLY LOW**	**CRITICALLY LOW**	**CRITICALLY LOW**

Domain	Cockle et al. (2017)
**1 – Do the research questions and inclusion criteria for the review include the PICO components?**	**YES**<br>Rationale: The PICO was identified in the objective (considered the research question) of the study and complies with the inclusion criteria established by the reviewers. The PICO structure was formally presented in the supplementary material.
**2 – Does the review report contain an explicit statement that the review methods were established before the review was carried out, and did the report justify any significant deviations from the protocol? (critical)**	**NO**<br>Rationale: The registration of the research protocol was not presented, and there was no mention of the research protocol.
**3 – Have the authors of the review explained the selection of study designs for inclusion in the review?**	**YES**<br>Rationale: Randomized clinical trials (RCTs) were the study designs of interest.
**4 – Did the authors of the review use a comprehensive literature search strategy? (critical)**	**YES**<br>Rationale: Four databases were used; the search strategies were presented in the supplementary material. A search was carried out in the gray literature, clinical trial registries, and references of previously published systematic reviews and meta-analyses.
**5 – Did the authors of the review select the studies in duplicate?**	**YES**<br>Justification: Two independent authors selected and collected the information of interest from the included studies, but this was not explicitly reported in the main text.
**6 – Did the authors of the review extract data in duplicate?**	**YES**<br>Justification: Two independent authors extracted the information of interest from the included studies, but this was not explicitly reported in the main text.
**7 – Do the authors of the review provide a list of excluded studies and justify the exclusions? (critical)**	**NO**<br>Justification: There is no list available that presents and justifies the studies excluded after full reading.
**8 – Do the review authors**	**YES**

**describe the included studies in adequate detail?**	Rationale: The study described the population, intervention, comparators, outcomes, and type of study. It also provided details of the population, intervention, and comparators (where necessary), reported the follow-up time of the included studies, and the outcomes of interest. These results are presented mainly in the supplementary material.
**9 – Did the review authors use a satisfactory technique to assess the risk of bias (RoB) in individual studies that were included in the review? (critical)**	**NO**
	Rationale: The methodological quality of the included studies was assessed, but there was no mention of a formal risk of bias assessment.
**10 – Did the authors of the review report the sources of funding for the studies included in the review?**	**NO**
	Rationale: The funding sources of the primary studies included in the review were not presented.
**11 – If a meta-analysis was carried out, did the authors of the review use appropriate methods for the statistical combination of results? (critical)**	**YES**
	Rationale: A systematic review with network meta-analysis was conducted to evaluate the comparative efficacy and tolerability of mepolizumab and omalizumab as complementary therapy to standard treatment in patients with severe asthma.
**12 – If a meta-analysis was carried out, did the review authors assess the potential impact of the risk of bias in individual studies on the results of the meta-analysis or other evidence synthesis?**	**NO**
	Rationale: The methodological quality of the included studies was assessed, but the potential impact of bias in these studies was not discussed.
**13 – Did the review authors consider the risk of bias in individual studies when interpreting/discussing the results of the review? (critical)**	**NO**
	Rationale: The methodological quality of the included studies was assessed, but the potential impact of bias in these studies was not discussed.
**14 – Have the authors of the**	**YES**

**review provided a satisfactory explanation and discussion of any heterogeneity observed in the results of the review?**	Rationale: The presence of heterogeneity among the studies was evaluated, and its potential influence on the results was discussed in the text.
**15 – If they carried out quantitative synthesis, did the authors of the review carry out an adequate investigation of publication bias (small study bias) and discuss its likely impact on the results of the review? (critical)**	**NO**
	Rationale: The text did not discuss issues related to possible publication bias.
**16 – Have the authors of the review reported any potential sources of conflict of interest, including any funding received to carry out the review?**	**YES**
	Rationale: The authors reported potential conflicts of interest and sources of funding.
**General methodological quality**	**CRITICALLY LOW**
